# Decomposing the molecular complexity of brewing

**DOI:** 10.1038/s41538-020-00070-3

**Published:** 2020-08-20

**Authors:** Stefan A. Pieczonka, Marianna Lucio, Michael Rychlik, Philippe Schmitt-Kopplin

**Affiliations:** 1 Chair of Analytical Food Chemistry, Technical University of Munich, Freising, Germany; 2 Research Unit Analytical BioGeoChemistry, Helmholtz Zentrum München, Neuherberg, Germany

**Keywords:** Agriculture, Small molecules

## Abstract

The compositional space of a set of 120 diverse beer samples was profiled by rapid flow-injection analysis (FIA) Fourier transform ion cyclotron mass spectrometry (FTICR-MS). By the unrivaled mass resolution, it was possible to uncover and assign compositional information to thousands of yet unknown metabolites in the beer matrix. The application of several statistical models enabled the assignment of different molecular pattern to certain beer attributes such as the beer type, the way of adding hops and the grain used. The dedicated van Krevelen diagrams and mass difference networks displayed the structural connectivity of the annotated sum formulae. Thereby it was possible to provide a base of knowledge of the beer metabolome far above database-dependent annotations. Typical metabolic signatures for beer types, which reflect differences in ingredients and ways of brewing, could be extracted. Besides, the complexity of isomeric compounds, initially profiled as single mass values in fast FIA-FTICR-MS, was resolved by selective UHPLC-ToF-MS^2^ analysis. Thereby structural hypotheses based on FTICR’s sum formulae could be confirmed. Benzoxazinoid hexosides deriving from the wheat’s secondary metabolism were uncovered as suitable marker substances for the use of whole wheat grains, in contrast to merely wheat starch or barley. Furthermore, it was possible to describe Hydroxymethoxybenzoxazinone(HMBOA)-hexosesulfate as a hitherto unknown phytoanticipin derivative in wheat containing beers. These findings raise the potential of ultrahigh resolution mass spectrometry for rapid quality control and inspection purposes as well as deep metabolic profiling, profound search for distinct hidden metabolites and classification of archeological beer samples.

## Introduction

The yearly worldwide consumption of beer adds up to 1.96 billion hectoliters (as of 2016). Thus, beer is, besides wine, the most consumed fermented alcoholic beverage. Brewing handicraft evolved in more than 5000 years from ancient brewers over the German purity law from 1516 to high scaled, modern industrial brewing^1^. The most recent developments, namely the “craft beer revolution”, refuses the trend of “macrobreweries” and emerge a multitude of smaller, more diverse brewhouses. Hops (*Humulus lupulus* L.), which are the main focus of experimentation, are one of the defining ingredients of beer. Besides that, (barley) malt, water and yeast contribute to the complex aqueous mixture of volatile and non-volatile molecules known as beer. The small molecules (<1000 Da) are referred to as the beer metabolome and play an important role in beer characteristics such as taste, aroma, yeast fermentation, foam stability, or beer ageing. The measurement of a group of chemically characterized and biochemically annotated metabolites is known as targeted metabolomics. Using different analytical methods such as GC- and LC-(ToF)-MS/MS allowed to characterize the phenols and polyphenols^2^, hops bitter acids^3^, the carbohydrates and their degradation during storage^4^, or their reactions with amino acids and proteins analyzing Maillard reaction markers^5^. Profiling specific volatile compounds in beer enable to show a difference between top and bottom fermenting yeasts^6^. In contrast, non-targeted metabolomics means a comprehensive analysis of all measurable analytes, including chemical unknowns^7^, achieving an optimal metabolome coverage^8^. It provides extensive datasets, which are used to explore novel features or characterize differences between samples using biostatistics, biochemistry, and informatics for data mining and interpretation^9,10^. By non-targeted metabolic profiling it is possible to differentiate beer types^11,12^, age groups^13^, origins^14^, different storing conditions^15^, color characteristics^16^, or hop varieties^17,18^ using high-resolution analytical methods. Profiling of volatile fingerprints of hops and barley^19^, yeast strains^20^, or different beer types^21^ was carried out by means of either headspace or bubbling burst (GC)-MS analysis. Besides mass spectrometry, nuclear magnetic resonance (NMR) spectroscopy was applied to beer analysis to differentiate beer types^22^, brewing sites^23^, raw materials, or influences on yeast fermentation^24^. The range of analyzed molecules, which characterize the differences of the samples, reaches from carbohydrates, amino acids, small organic acids over bitter acids, (poly)phenols and purines to more volatile terpenes, esters, alcohols, aldehydes, and ketones. One major drawback of non-targeted metabolomics is the dependence on and limitation to database annotations. The outnumbering unknown signals often referred to as “molecule features” are not characterized.

Non-targeted metabolic profiling can exceedingly benefit from a promising mass spectrometric method in beer analysis, the Fourier transform ion cyclotron resonance (FTICR) mass spectrometry. Gougeon et al.^25,26^ already described the chemical space of wine by a direct flow-injection ESI-method coupled to the FTICR instrument. It was shown that this approach has the power of resolving not hundreds, but thousands of molecules in a short time. Indeed, Fourier transform mass spectrometry techniques are the most advanced mass analyzers concerning mass accuracy and resolving power. The unrivaled mass resolution enables a Flow-injection-analysis approach, which gives access to compounds of a wide polarity range. Due to ultrahigh resolution (~500,000 res. power at *m*/*z* 400) and accurate mass measurement (~0.1 ppm) FTICR-MS can separate and assign a molecular formula to each signal, providing information about the (bio)chemical class of these often yet unknown analytes. As thousands of features can be characterized it provides universal information about the analyzed samples that remain hidden otherwise. Furthermore, by connecting marker substances by mass difference networks^27^ and displaying patterns of chemical compositions in van Krevelen diagrams^28^, it is possible to infer the markers’ compositional nature. These visualization methods allow us to make well-sustained assumptions of molecule groups, which differentiate diverse samples. As a result, the disclosed metabolic signature of unknown samples can be recognized and assigned. Specific compositions, which are essential for characterizing certain metabolic profiles, can be perceived by statistical evaluation of rapid and holistic FTICR measurements. However, FIA-FTICR-MS lacks information about isomers and concrete molecular structures, which requires a second analytical technique. Tandem UHPLC-ToF-MS is able to resolve isomeric compounds and provide deeper structural information. Based on the exact *m*/*z* values found in FTICR, the fragmentation of dedicated compounds and isomers enables identification of the most significant molecules on a structural level. The presented approach closes a gap between the availability of a huge multitude of analyzed features, their compositional annotation and deep structural information. It opens the application for the recognition of the metabolic signatures and the profound search for distinct hidden metabolites.

## Results

### Visualization of the molecular complexity

**Fig. 1:**
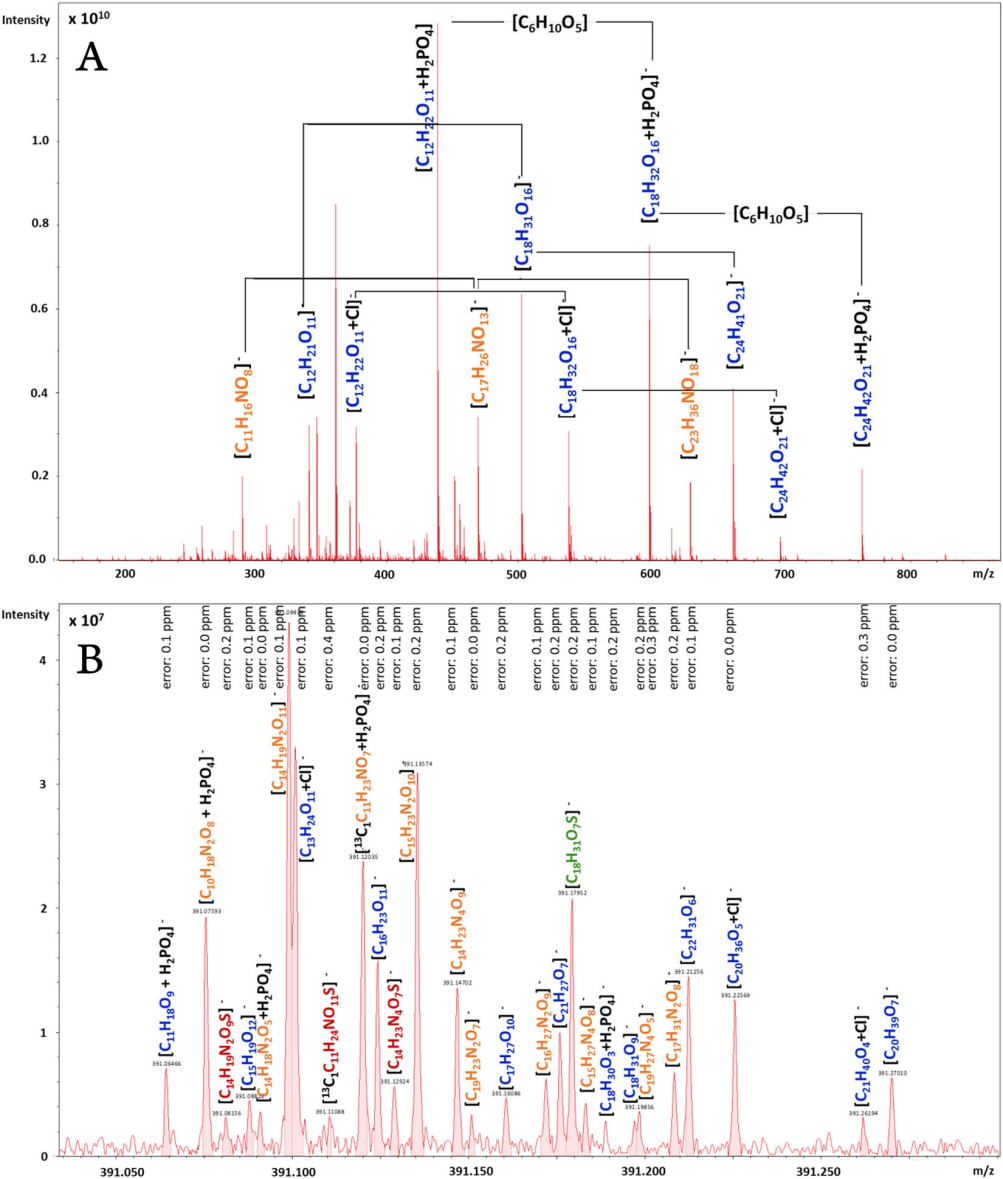
FTICR-MS spectrum reveals the chemodiversity of a Pilsner beer and biochemical patterns therein. The full-scale view (**a**) shows hexose condensation patterns and an excerpt of the nominal mass *m/z* 391 (**b**) illustrates the resolved chemodiversity of the beer inside one single nominal mass. Annotated sum formulae and mass errors are given above the mass peaks. Color code of the sum formulae: CHO blue; CHNO orange; CHOS green; CHNOS red. Adduct formation is expressed by +H_2_PO_4_ for dihydrogenphosphate and +Cl for chloride, respectively.

**Fig. 2:**
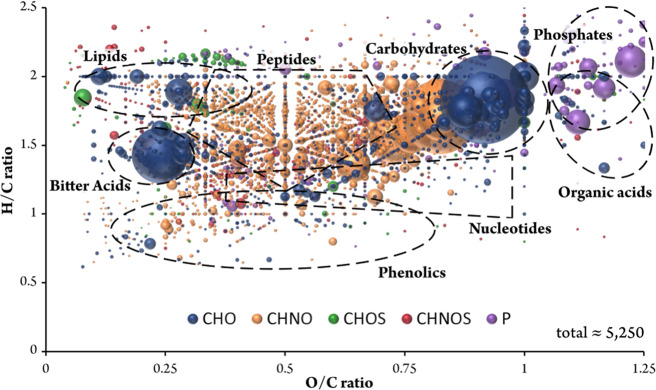
Van Krevelen diagram (H/C vs. O/C) of beer compositions shows their diversity and associated compounds classes. Annotations, which appear in at least 5% of all beer samples are shown. Areas specific for certain compound classes are marked with dotted lines. Color code: CHNO blue; CHNO orange; CHOS green; CHNOS red; P violet; Cl light violet. The bubble size indicates the mean relative intensities.

A diverse set of 85 bottled beers from different countries and of different types was profiled as the first batch. To explore the compositional diversity and molecular complexity of each individual beer the samples were analyzed by flow-injection ESI(−) FTICR-MS. The chemical space of beer is as diverse as the variety of different raw materials and their treatment during the brewing process including malting, roasting, boiling, fermentation, and filtration. As an example, Fig. 1 shows the spectrum of a Pilsner beer. The macroscopic general view (Fig. 1a) shows the abundancy of (oligo)saccharide patterns. However, the detailed view of a single nominal mass (Fig. 1b) revealed up to 27 *m/z* values within the mass of 391, which could be assigned to molecular formulae with a mean error of <0.1 ppm (<1/10 of an electron mass, respectively). The molecular variety of the beer samples, which ranges from peptides [C_19_H_28_N_4_O_5_], carbohydrates [C_13_H_24_O_11_], fatty acids [C_21_H_40_O_4_] through their sulfates [C_18_H_31_O_7_S] to isotopologues of potential Maillard reaction products like desoxyfructosyl(iso-)leucine [^13^C_1_C_11_H_23_NO_7_], could be displayed in one single nominal mass by highly resolved FTICR measurements. In total, an average of 2800 compositions could be found in each beer spectra. Bearing in mind that distinct isomers exist for a given formula, the 27 molecular formulae in the spectrum excerpt represent 68 hits reaching from 0 to 11 isomers in common databases. Therefore, the FIA-FTICR-MS spectrum of a single beer can be considered as an instantaneous overview of several thousands of compounds present in various concentrations. All *m*/*z* values assigned to a molecular formula and present in at least 5% of all beer samples are depicted in a two-dimensional van Krevelen diagram (Fig. 2). Thereby the masses can be associated to chemical families like carbohydrates, peptides, organic acids, phenolics, lipids, nucleotides or even hops bitter acids and their corresponding derivatives^29^. Plotting in the van Krevelen diagram the 350 formulae, which were present in over 95% of the beers spectra, we can recognize that the beer matrix seems, in general, to be defined by carbohydrates and derivatives, peptides, but also the hops bitter acids. In contrast to this, lipids and phenolic compounds were more specific for the single beers or group of beers (Supplementary Fig. 1).

**Fig. 3:**
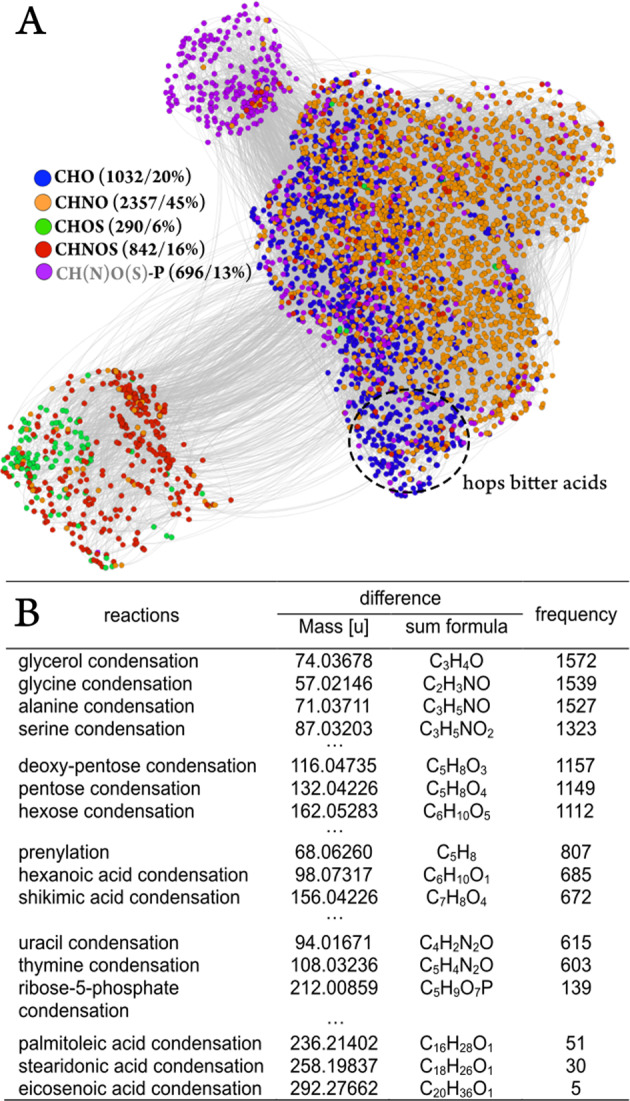
Mass difference network of the beer samples’ annotations and their (bio)chemical connectivity. Chloride adducts were converted into their dedicated [M–H]^−^ ions in silico. Color code compare Fig. 2. The area of hops bitter acid derivatives inside the mass difference network (**a**) is marked. An excerpt of (bio)chemical reactions with their dedicated mass and sum formula differences and the frequencies they occur in the network is given below (**b**).

By displaying assigned elemental formulae in a mass difference network^27^ one can exploit the exact mass information provided by FTICR-MS and set the CHO, CHNO, CHNOS, CHOS, and P chemical spaces into relation. Figure 3a shows that the sulfur containing spaces were separated from a highly connected CHO/CHNO sphere. The same holds true for phosphate containing molecules, which were mostly connected to the other spaces by glycerolphosphate, phosphoethanolamine, hexosephosphate, and phosphorylation itself. Mass differences indicating mainly reactions with amino acids were the most dominant inside the CHNO chemical space and between CHO and CHNO spaces (~50%). Condensation of hexose and pentose species are the most abundant sugar-related reactions connecting (oligo)saccharides with their dedicated aglyca. Reactions regarding more specific metabolic pathways like prenylation (terpenoids) could be found besides the condensation of nucleic bases and glycerol. Overall, raw chemical-related reactions (roasting/malting/boiling) were represented on a par with biochemically driven reactions (raw material/fermentation). An extract of the frequencies of individual modifications can be found in Fig. 3b.

### Multivariate analysis

**Fig. 4:**
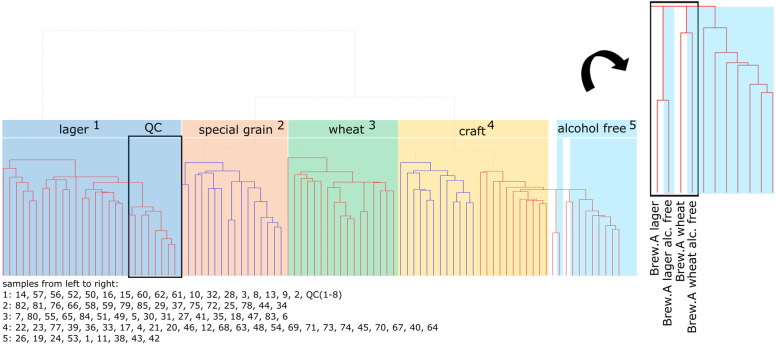
Hierarchical clustering arranges the beer samples’ FTICR mass spectra with regard to their beer type. Color code of the observed clusters: lager beer blue; beer brewed with special grain red; wheat beer green; craft beer yellow; alcohol-free beer light blue. The cluster of QC lager beer samples is framed. The enlarged excerpt shows the cluster of one brewing site’s alcohol containing and alcohol-free beers. The samples’ order is stated below.

The hierarchical clustering analysis (HCA) showed a general overview of the similarities across the different samples revealed a cluster of typical lager beers samples (Fig. 4). The quality control samples, namely aliquots of one same lager beer, were correctly located in exactly this group and build an own sub cluster, which showed that the fingerprint of this beer is conserved through the different batches. Beers with special grains like roasted malt, oat, or gluten-free grain were grouped together as well as wheat beers and alcohol-free beers. Besides these clusters there was a group mainly but not exclusively consisting of craft beers and special Belgian beers. Some more conventional beers were also allocated inside this group, probably due to the overlap of specific molecular patterns. A detailed inspection of the dendrogram plot revealed two pairs of beer from one brewery (denominated “brewery A” in the following)—namely the brewery A’s lager and wheat beer with their corresponding alcohol-free versions. These pairings reflect the fact that the dealcoholization process in this brewery consists mainly of downdraft evaporation of the original alcohol containing beer. The brewing process itself stays the same, which makes these beers very similar.

### OPLS-DA model 1: beer type

**Fig. 5:**
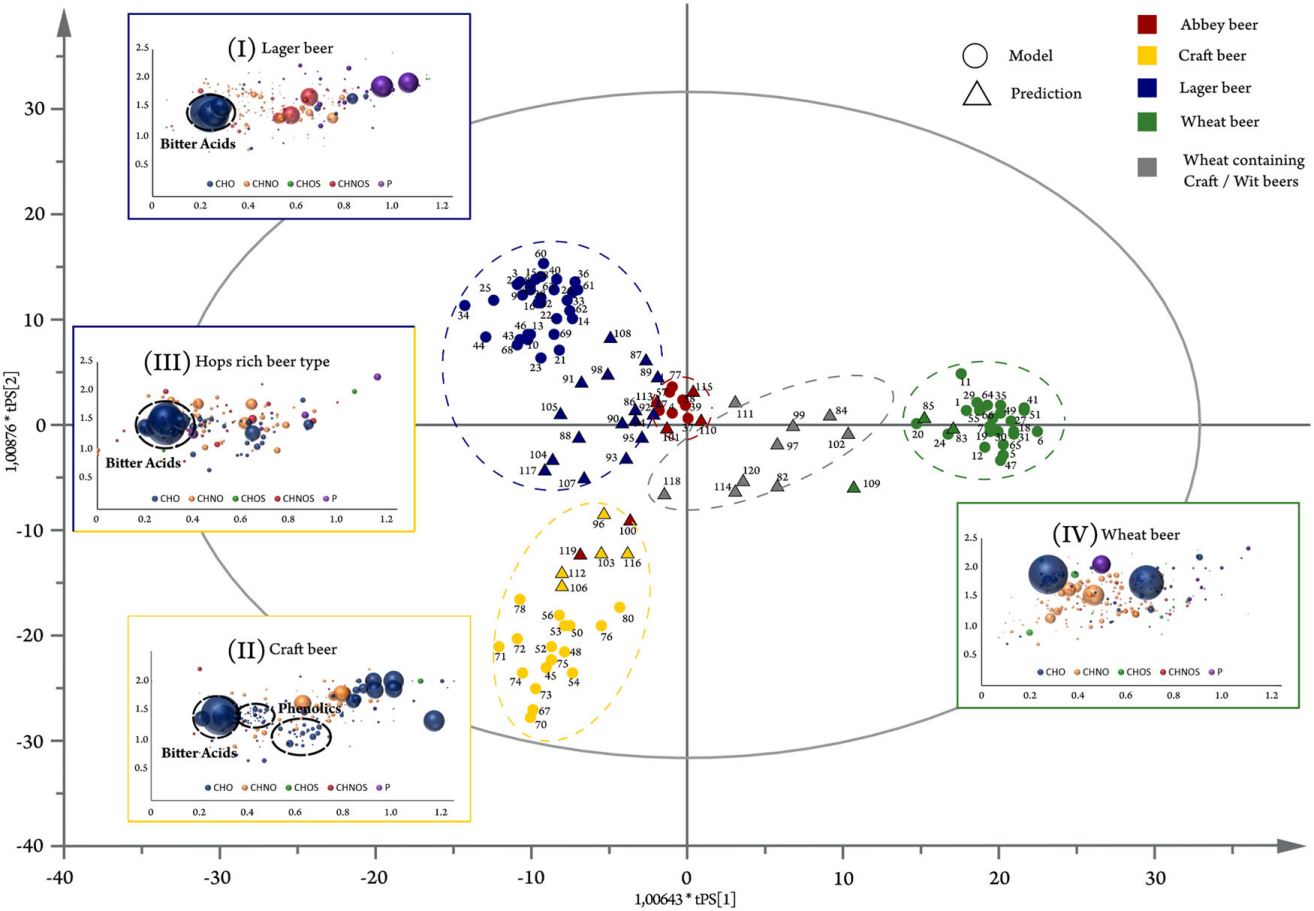
OPLS-DA model’s score plot for the beer-type observation. The score plot is surrounded by the different observations’ van Krevelen diagrams (lager beers (I); craft beers (II); rich hopped beer types (III); wheat beers (IV)). Color code and bubble size compare Fig. 2. Samples included in the model calculation are depicted as circles, whereas predicted samples are represented as triangles. Craft and lager beers are summarized as hops rich beer types to reflect the separation of metabolites in the first component.

The first OPLS-DA model distinguishes between the different beer types (Fig. 5). Wheat beers were separated from the other beer types in the first component (*x*-axis). In the orthogonal second component (*y*-axis) it was possible to differentiate between classical lager beers and craft beers. The fourth class, the traditional Belgian abbey beers, were located in the middle of the score plot, whereas the spontaneous inoculated geuze beers were excluded from the model as outliers. The detailed statistical (Supplementary Table 1), loading plots (Supplementary Fig. 2), and score-plot coordinates (Supplementary Table 2) for each model are given in the supplementary information.

**Fig. 6:**
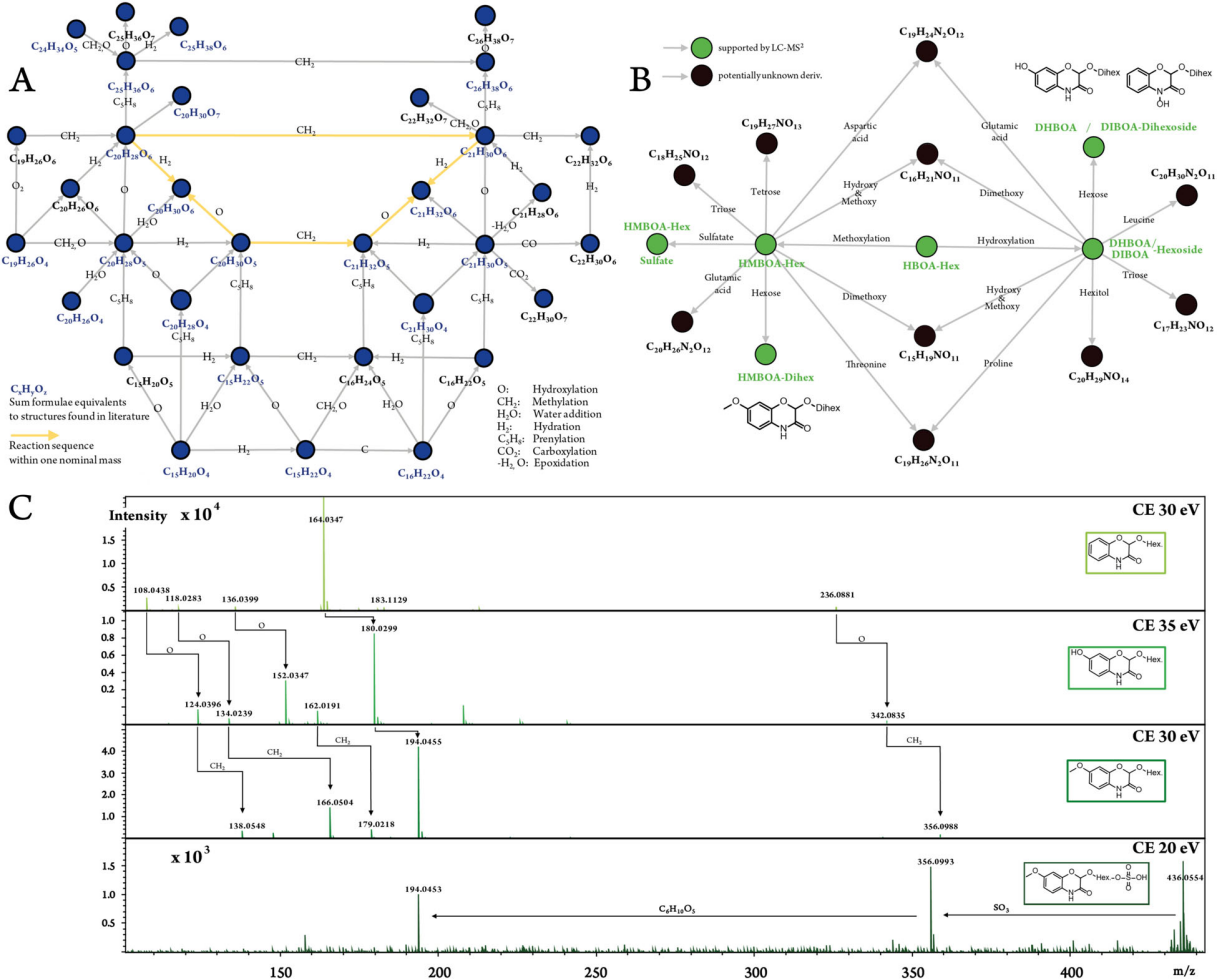
Detailed excerpts of the mass difference networks for selected hops rich beer-type markers and wheat grain markers. The nodes represent the annotated ions with given sum formulae or molecule names. They are connected by edges representing the sum formula differences for the hops rich beer-type markers (**a**) and the biochemical reaction for the wheat grain markers (**b**), respectively. All nodes depicted are considered marker substances. Wheat grain markers are additionally characterized by UPLC-MS^2^ of wheat beer sample 41 with literature matching retention time order and MS^2^-spectra showing respective fragmentation and mass difference pattern (**c**)^32,39^.

The first component revealed the most significant molecular pattern separating wheat beers from the lager and craft beers. Both the latter beer types feature a great amount of hops compared with wheat beers and thus can be denominated “hops rich beer types”. The masses with the most negative loadings reflected this characteristic of a strong hops profile. The Van Krevelen diagram of their formulae showed a specific cluster of CHO-molecules in the region of 0.2 < O/C < 0.4 and 1.2 < H/C < 1.6, respectively (Fig. 5i). As mentioned before, this area of the diagram is typical for terpenoids and more accurately hops bitter acids (terpeno-phenolics) in the beer matrix. This pattern was also observed in the mass difference network, showing an agglomerated cluster of hop-rich beer-type markers in a certain area (Fig. 3a). The annotation of the given masses in databases offered exactly those hops bitter acids. Therefore, it is possible to uncover the area of the mass difference network, where the chemistry of the hops bitter acids is located. A number of 58 marker substances for rich hopped beers could be determined as derivatives by their molecular formula, whereas only 20 of them (35%) were found to have equivalent structures in the databases and pertinent literature (Supplementary Table 3). As FTICR-MS is not capable of distinguishing isomers, the [C_21_H_30_O_5_] marker can represent humulone, adhumulone or iso-humulone, but most likely a mixture. Further already known precursor molecules like prenylphlorisobutyrophenone [C_15_H_20_O_4_] and prenylphlor-isovalerophenone [C_16_H_22_O_4_] as well as bitter acid derivatives like cohumulone [C_20_H_28_O_5_], deoxycohumulone [C_20_H_28_O_4_], dihydrohumulone [C_21_H_32_O_5_], or humulinone [C_19_H_26_O_5_] are surrounded by molecular formulae without suitable hits (Fig. 6a). A demethylation reaction of the potential cohumulinone [C_20_H_28_O_6_] leads to the molecule [C_19_H_26_O_6_], whereas a decarboxylation of [C_20_H_30_O_7_] leads to humulone [C_21_H_30_O_5_]. Overall, finding literature equivalents of oxygenated structures like [C_19_H_26_O_6_], which might indicate hydroxyl-, epoxy-, carboxy-, or peroxyderivatives, turned out considerably difficult. Furthermore, reduction/hydration and addition/elimination of water seem to be important reactions inside this excerpt network of marker substances. Pairs of marker molecules within the same nominal mass (e.g., C_20_H_28_O_6_/C_21_H_32_O_5_; C_19_H_26_O_6_/C_20_H_30_O_5_; C_20_H_26_O_6_/C_21_H_30_O_5_) underlined the necessity of high resolving analytical techniques. In the second component, more oxygenated bitter acid species as well as phenolic and polyphenolic compounds and their dedicated glycosides seem to be characteristic for craft beers due to the typical dry hopping process (Fig. 5ii).

It was possible to confirm the calculated profiles of the beer types by the vicinity of the different types between the model and prediction sample sets (Fig. 5). Only the position of two samples in the score plot defy the cluster. Sample numbers 100 and 119, both brewed in a certified abbey and, therefore, characterized as abbey beer, were located inside the craft beer region. Besides this origin, the actual brewing technique of these beers is described as amber ale and triple ale, both in agreement with craft beer styles including dry hopping. Therefore, not the brewing location itself, but the molecular signature of the brewing process stands in the foreground. A second group of beers, that could not be assigned precisely, were craft beers brewed with wheat and Belgian wit beers made with raw wheat. These beers share the signature of craft beers (ale yeast; preferably strongly hopped) and the signature of wheat beers (wheat grain as ingredient), for which reason they were located between those beer types. The organic wheat beer (sample 109) differed slightly as well. These findings suggested that the compounds with the most positive loadings define the molecular pattern of wheat. For the investigation of specifically the wheat signature a second model was created.

### OPLS-DA model 2: grain

The second OPLS-model was created to extract the influence of the ingredient wheat on the beer’s metabolome (Supplementary Fig. 3). All beers brewed with some amount of wheat were defined as wheat containing beers, regardless of their beer type and other brewing parameters. These stood against beers brewed exclusively with barley. Notwithstanding, that the model sample set consisted of beers with a plurality of various characteristics, it was possible to perform the separation based on the grain used without any ambiguous assignments. In addition to the intended separation it could be remarked that the wheat containing craft beers (sample 53, 54, 73), which were brewed with ale yeasts and dry hopped, were separated by the orthogonal information by the second component (*y*-axis). In the loading plot, several highly significant wheat grain markers, such as [C_14_H_17_NO_8_], [C_14_H_17_NO_9_], and [C_15_H_19_NO_9_], are separated by simple biochemical reactions (e.g., hydroxylation; methylation) and most likely belong to the family of benzoxazinoid hexosides. The intensity distribution of the mentioned markers is given in the supplementary information (Supplementary Fig. 4). These compounds are described to be specific phytoanticipines for wheat^30^ compared with barley and partially described in wheat beer^31^. Again, an excerpt of the mass difference network of the wheat marker substances revealed six masses corresponding to benzoxazines, which were already characterized by de Bruijn et al.^32^, and a plurality of potential derivatives (Fig. 6c). Against this background, the sulfatation reaction of the HMBOA-hexoside to the respective sulfate appeared especially promising. These secondary metabolites and their dedicated derivatives seemed to be a crucial part of the metabolic signature of wheat containing beers.

The prediction model (Supplementary Fig. 3) showed that the typical German wheat beers containing malted wheat were as well recognized as the Belgian wit beers, which contain unmalted wheat. In contrast, the metabolic pattern of the wheat grain in wheat containing craft beers (sample numbers 100, 114, 118) was recognized less strongly. The comparatively low amount of wheat was opposed by the contrary heavy hops signature. For beers brewed with merely wheat starch no wheat signature could be observed. These findings confirmed the applicability of the calculated pattern and advice to identify certain specific marker substances to detect even low amounts of wheat metabolites.

### UHPLC-ToF-MS: marker identification

To support the interpretation of the FTICR-MS data and verify the predicted structures, we performed UHPLC-ToF-MS^2^ measurements on selected samples. The marker substances for a rich hops profile and the wheat metabolome were investigated in depth.

The marker substances of beers with a rich hops profile in the Van Krevelen region of 0.2 < O/C < 0.4 and 1.2 < H/C < 1.6, respectively (Fig. 5i), were proposed as hops bitter acid derivatives. The UHPLC-MS measurements of a hops rich beer revealed mass traces fitting to 46 of the 58 sum formulae (80%) of the mentioned markers (Supplementary Fig. 5). This is a notably high rate because only 35% of the markers were found to have structural equivalents in mentioned databases or cited pertinent literature (Supplementary Table 3). Moreover, the LC-dimension gave a better idea of how complex the structures behind these masses are as up to 21 peaks could be found for one single formula, all being eluted in the chromatogram region, where hops bitter acid derivatives were found (3.5–7.0 min). The 22 detected isomeric compounds for humulinone [C_21_H_30_O_6_] stood in contrast to other formulae like [C_19_H_26_O_4_] (cohulupone), which were represented by only one chromatographic peak (Supplementary Fig. 5). By tandem mass spectrometry we were able to identify twelve hops bitter acid derivatives like cohumulinic acid [C_14_H_20_O_4_], hulupinic acid [C_15_H_20_O_4_], cohulupone [C_19_H_26_O_4_], (ad)humulone[C_21_H_30_O_5_], tricyclocohumol [C_20_H_30_O_6_], or tetracyclohumol [C_20_H_30_O_6_] on level two^33^ by comparison of fragmentation patterns and intensities with literature data (Supplementary Table 4). Opposing a wheat beer, which does not feature a rich hops profile, shows, that the corresponding mass traces are decisively higher in hops richer craft and lager beers verifying their discriminating character (Supplementary Fig. 5). It is worth noting that more than 100 MS^2^ spectra did not lead to hits in databases or literature, and therefore are considered level 3 identifications (Supplementary Table 5).

Benzoxazinoidic phytoanticipines of the wheat plant were proposed as specific wheat grain markers in the beer matrix (Fig. 6c). Again, the marker formulae of the FTICR-MS models were transferred into a preference list to selectively acquire tandem mass spectrometric spectra. By comparison with literature known MS^2^ fragmentation, eight HBOA-derivatives could be identified in wheat beer (level 2) (Supplementary Table 6). The retention time sequence of the HBOA-, DHBOA-, DIBOA-, and HMBOA-hexoside coincides with the one described by de Bruijn et al.^32^, whereas the predicted HMBOA-hexosesulfate was eluted earlier than the corresponding hexoside due to the polar sulfate group. The MS^2^-spectra of the monohexosides are compared in Fig. 6c. The cleavage of the hexose group from the HBOA-hexoside (1) [M–H]^−^-ion [C_14_H_16_NO_8_] results in an *m/z* value of 164.0348 [C_8_H_6_NO_3_]. The additional hydroxygroup of the DIBOA-hexoside (2) leads to the 180.0299 *m/z* ion [C_8_H_6_NO_4_]. Replacing the hydroxygroup by a methoxygroup, the *m/z* value of 194.0455 [C_9_H_8_NO_4_] can be found for the HMBOA-hexoside (3). The same pattern holds true for the 136.0399 [C_7_H_6_NO_2_], 118.0283 [C_7_H_4_NO], and 108.0438 [C_6_H_6_NO] fragment ions of the HBOA-hexoside. It was not possible to extract complex fragmentation pattern of the HMBOA-hexosesulfate (4) as it was a minor component with a peak intensity about 30 times lower than the respective hexoside. However, the loss of the sulfate group from the quasi-molecular ion 436.0554 [C_15_H_18_NO_12_S] to the dedicated HMBOA-hexoside (3) [M–H]^−^-ion 356.0993 [C_15_H_18_NO_9_] could be observed. Hereupon both compounds share the loss of the hexose sugar. The dihexoside DHBOA-, DIBOA-, and HMBOA-equivalents showed several closely eluting isomeric peaks and were detected with lower retention times as they are more polar. All the substantiated compounds were only observed in wheat beer and none of them is present in beer exclusively brewed with barley, which confirms the assumption that benzoxazinoidic phytoanticipines are suitable specific compounds for the use of wheat grain. To our knowledge, the existence of a HMBOA-hexosesulfate has not been described before. However, for definite identification the synthesis of a corresponding standard would be needed.

## Discussion

Many studies have been published in the literature about beer metabolome analysis employing LC- and GC–MS either with time-of-flight or orbitrap instruments. The use of high-field Fourier transform ion cyclotron mass spectrometry is shown here for the non-targeted metabolic profiling of a diverse set of beer samples and enables a flow-injection analysis due to the ultrahigh resolution provided. We were able to demonstrate the benefits of the superior mass accuracy paired with the annotation in compositional networks. Constructing compositional mass difference networks exploits the exact mass information provided by FTICR-MS and enabled coverage of complex formulae and the whole compositional space. Thereby it was possible to assign molecular formulae like [C_29_H_35_N_5_O_10_S] to an exact mass likely corresponding to an Asp-Asp-Phe-Phe-Cys peptide or [C_10_H_14_N_5_O_8_P] to guanosinemonophosphate (GMP). Even at low masses (*m*/*z* 362.05072 for GMP) over 10 formulae are valid inside a 3 ppm window (Supplementary Fig. 6). By the provided mass error window of 0.1 ppm (0.002 ppm for GMP) and the possibility to resolve isotopic fine structure we could ensure correct annotations with our FIA-FTICR-MS approach. It allows us to directly proceed from *m/z* values to the compositional space, depict thousands of yet unknown structures and assign their structural family concerning their position in the van Krevelen diagram and connectivity inside the mass difference network. Respective patterns were found for hops bitter acids and biochemical connectivity of blepharine derivatives.

By supervised OPLS-DA modeling, we were able to extract the profound metabolic signature underlying different beer types within the brewing process. The classification power of the models was highly significant. The *p*-values (calculated after the CV-ANOVA) were lower than 2E−19; such values bring us to exclude possible overfitting. Both models exceed *Q*^2^ values of 0.6, for the quality of prevision, and *R*^2^*Y* values of 0.95, for the goodness of the fit, proving their statistical relevance^34^. The molecular signatures for both lager and craft beers were dominated by the quantity of hops used. However, lager beer is predominantly brewed with hops varieties, which are rich in bitter acid compounds. Confirmatory, humulone and cohumulone isomers and derivatives appeared as marker masses for these types of beers. By the analysis of the mass differences of masses dedicated to precursors and intermediates even the whole biosynthesis of these typical hops metabolites could be traced inside the beer matrix. The position of discriminating compositions in the Van Krevelen diagram showed, that more oxygenates bitter acid species as well as phenols, polyphenols, and dedicated glycosides are characteristic for craft beers. Dry hopping of aroma hops after the boiling or fermentation process, which is typical for this type of beers, adds a multitude of mentioned compounds to their dedicated metabolic profile due to ethanolic extraction of the hops^35^. Moreover, adding hops umbels to the wort or young beer represents a heavy input of oxygen, which advances oxidation processes. Hydrolysis, (de-)hydration, epoxidation, peroxidation, and cyclisation mechanisms of hops compounds, which also lead to an altered bitterness perception, are known and described in literature as well as the presence of phenolic acids, coumarins, flavonoid polyphenols, and their glycosides^36^. However, the immense compositional complexity, which evolves from these reactions and is addressed in this work, still needs to be discovered. Duarte et al.^37^ already suggested using ^1^H-NMR that the main difference between lager and craft beers in terms of the metabolome can be traced back to aromatic compounds. The prevision of a sub-sample set confirmed the universal applicability of our model and strengthen the fact that we were able to differentiate type and parameters of brewing. Both beer types are commonly brewed without wheat. Therefore, the wheat metabolome was assumed to be an important discriminating and defining factor for wheat beers. ^1^H-NMR analyses^22,38^ again held aromatic compounds responsible for the differentiation of grains. A statistical model opposing the grains used was established to tackle the challenge of a comprehensive description of the wheat metabolome and eventually gain access to structural information. The fact that some beers share the molecular signature of hops and wheat (see prevision of the first OPLS-DA model) and the circumstance that wheat beers are to different extents brewed with barley malt as well, made this step of a second model essential.

The occurrence of blepharine derivatives as secondary metabolites derived by the wheat grain as marker substances in beer emphasizes the holistic and deep nature of our profiling approach. Hydroxybenzoxazinone (HBOA) and its derivatives are known to be phytoanticipines with antifungal, antimicrobial and insecticide properties in the wheat plant^30^. Blepharines are stored in the vacuole and activated following cell damage through β-glucosidase activity^30^. It may be anticipated that the described sulfate plays a role in either storage or transportation of the phytoanticipines. It was previously shown that the phytoanticipines are modified during food processing and fermentation^39,40^. Thus, chemical reactions during malting and boiling may also contribute to the multitude of possible derivatives yet unknown in the wheat and beer matrix. Compositional networks provided access to new metabolites even in the beer matrix, which makes a similar approach inside the wheat plant, grain or its compartments especially promising. Worth mentioning is the fact that no signature of wheat metabolites could be found in FTICR- and LC-ToF-MS measurements with regard to beers merely brewed with wheat starch. These beers lack the secondary metabolome of the wheat grain. The combination of analytical and statistical techniques presented here raise the potential of substantial advances in yet open questions regarding both brewing science and industry. In total, the metabolic profile of beer type and grains provided by FIA-FTICR-MS could be verified by the identification of 18 (level 2) and 118 (level 3) compounds, respectively, for the signature of rich hopping and the use of wheat. As an outlook, the potential of ultrahigh resolution for food inspection or quality control applications is shown by the differentiation between beers brewed with wheat and merely wheat starch. Ongoing work focuses on expending the approach toward other descriptive parameters and archeochemical application of the presented metabolic signatures. Archeochemical investigations on wines and beers are generally executed by GC–MS-^41^ and IR-based^42^ measurements or are restricted to targeted approaches^43^. The presented metabolic profiles in future will be beneficial in deep profiling of ancient beer-like beverages and beers of the earlier modern era.

## Materials and methods

### Beer samples

A total of 85 samples of bottled beers produced in different countries were analyzed. They range from common lager or wheat over craft and abbey to lacto fermented geuze beers. Light and dark, top and bottom fermented, filtrated and non-filtrated, organic and gluten-free samples with alcohol contents of 0–12% are covering the whole variety of purchasable beers in close to any possible combination. Thereby the most comprehensive mapping of beers’ metabolome and prevention of covarying metadata was achieved. The samples were purchased at local grocery stores in December 2017 and stored at −20 °C prior preparation for analyses. A second independent sample batch, which includes 35 beers, was purchased in 2019 and used as prediction and validation set. The beer specifications are summarized in the supplementary information (Supplementary Table 2).

### FIA-FTICR-MS measurements

High-resolution mass spectra were acquired on a Bruker solariX Ion Cyclotron Resonance Fourier Transform Mass Spectrometer (Bruker Daltonics GmbH, Bremen, Germany) equipped with a 12 Tesla superconducting magnet (Magnex Scientific Inc., Yarton, GB) and a APOLO II ESI source (Bruker Daltonics GmbH, Bremen, Germany) operated in negative ionization mode. The negative ion mode was preferred based on greater variety in the composition, abundance of compounds and a smaller number of suppressing adducts with respect to heavy potassium adduct formation in the positive ionization mode. The beer samples were injected once into the microelectrospray source diluted 1:500 in methanol and the total analysis time of a sample was 10 min. The used reagents, sample preparation, and instrumental parameters are given in Supplementary Table 7. Possible space charge effects were recalibrated by mass difference mapping^44^. The samples were measured over a period of 18 months in randomized order using a representative lager beer as quality control. Mass accuracies reached values lower than 0.1 ppm between and within measurement days. Furthermore, the conservation of the ion intensities and the molecular fingerprint could be observed by this approach (see data-mining HCA).

### FTICR-MS data processing and visualization

The FTICR spectra were exported to peak lists with a cut-off of signal-to-noise ratio (S/N) of 6 using the DataAnalysis 4.2 software. Only singly charged ions were included. Processing and filtration of the peak lists (FT-side loops and isotopologue filtering) were performed by an in-house R-based software tool on basis of single spectra. Peak alignment was performed within a threshold of 1 ppm. Thereby an overall matrix of 13,800 masses was created. To obtain molecular formulae, the exact masses were subjected to mass difference network (MDN) analysis using the NetCalc software tool^27^. The network calculation was repeated ten times and coinciding formula assignments were kept, which led to approximately 10,500 unambiguous molecular formulae in the C, H, N, S, O, P, Cl space. The developed mass difference network, in which nodes represent molecular formulae and edges represent chemical reactions, was visualized by the open accessible Gephie Viz Platform^45^ using the open order algorithm. The masses with a frequency below 5% through all the samples were not considered during further data mining. Small mass transitions like oxidation, methylation, hydrogenation, or amination were withheld for visualization due to computing power. Van Krevelen diagrams were chosen to associate annotated *m*/*z* values to chemical families based on the procedure illustrated by Schmitt-Kopplin et al.^29^. Library searches were executed using an R script based on the MassTRIX approach^46^ including the Human Metabolome Database (HMDB)^47^, the Chemical Entities of Biological Interest (ChEBi)^48^, Metacyc^49^, Lipid maps^50^, the Yeast Metabolome Database (YMDB)^51^, and an in-house peptide database consisting of all in silico peptides.

### UHPLC-ToF-MS measurements and structural identification

The beer sample 52 (hops rich craft beer) and sample 41 (wheat beer) were analyzed in a fivefold concentration on a time-of-flight mass spectrometer (maXis, Bruker Daltonics, Bremen, Germany), coupled to an UHPLC system (Acquity, Waters, Eschborn, Germany). The preference list for fragmentation was compiled based on the substances’ masses, which occurred as marker for the hop rich beer types (sample 52) and wheat grain (sample 41) observations (Supplementary Tables 4–7). Further instrumental parameters are given in Supplementary Table 7. The search for comparable tandem mass spectrometric data was executed using the MassBank of North America^52^ and in silico fragmentation by MetFrag^53^ based on the KEGG^54^, HMDB^47^, and YMDB^51^ databases. Spectra were checked in mentioned literature source. The level of identification was assigned based on the criteria given by Sumner et al.^33^.

### Statistical analyses

The dataset, divided into a first batch defining the model and a second batch used for prevision and validation, was analyzed with different multivariate techniques. First, we used an unsupervised technique to cluster the different beer samples. The intensities were normalized (*z*-scores) and the clustering was calculated by using the average group linkage and the Pearson correlation coefficient for the distance measure (Hierarchical Clustering Explorer tool; HCE, 3.0). Afterward, the dataset was analyzed by different classification models applying supervised orthogonal partial least-square discriminant analysis (OPLS-DA). The Hotelling’s T^2^ test (95%) was applied to prohibit the influence of strong outliers on the models. For both the beer type and grain model it was possible to extrapolate the most discriminant features (*m/z* values). The lists of the most important masses were defined choosing the highest loadings values. The top characteristic masses were selected within the 95th percentile (264 masses for each class). The goodness of the fit and of the prediction were evaluated with the *R*^2^ and *Q*^2^ values. To exclude overfitting, we provide the *p*-value of the cross-validation analysis of variance (CV-ANOVA). In addition, based on the robustness of the classification models we could use them to make a prevision of a second sample set. The recognition of molecular pattern for the independent samples and thus the localization of those in the score plot could verify the universal applicability of the models. Those elaborations were done in SIMCA 13.0.3.0 (Umetrics, Umeå, Sweden). The marker formulae were depicted in van Krevelen diagrams for each class. By plotting H/C versus O/C atomic ratios it is possible to depict common compositional patterns within observations’ markers^25,28^.

### Reporting summary

Further information on research design is available in the Nature Research Reporting Summary linked to this article.

## Supplementary information

Supplementary information

Reporting Summary

## Data Availability

The datasets generated during and/or analyzed during the current study are available from the corresponding author on reasonable request.

## Code availability

The custom code and algorithms used during the current study are available from the corresponding author on reasonable request.
