# Supplementary material for: Decomposing the molecular complexity of brewing
Source: NPJ Sci Food. 2020 Aug 20;4:11. doi: 10.1038/s41538-020-00070-3 (PMC7441322; doi:10.1038/s41538-020-00070-3)
Supplement: Supplementary file 1 — Supplementary information [file 41538_2020_70_MOESM1_ESM.pdf]

Supplementary information

Decomposing the molecular complexity of brewing

Stefan A. Pieczonka,[a,b] Marianna Lucio,[b] Michael Rychlik,[a] Philippe Schmitt-Kopplin[a,b,*]

[a] Chair of Analytical Food Chemistry, Technical University of Munich, Freising, Germany
[b] Research Unit Analytical BioGeoChemistry, Helmholtz Zentrum München, Neuherberg, Germany
*corresponding author: schmitt-kopplin@helmholtz-muenchen.de

Table of Contents

Tables 2

Supplementary Table 1. Overview of the OPLS-DA models (exclusions, predictions) and statistical parameters(R^2Y, Q^2, VC-ANOVA) 2

Supplementary Table 2. Overview (beer type, grain used, OPLS-scores, set type) of measured samples' characteristics. 3

Supplementary Table 3. Tentative annotations of selected hops rich beer types markers on basis of sum formulae 6

Supplementary Table 4. Structural substantiation of hops rich beer types marker masses by means of UHPLC-ToF-MS^2 7

Supplementary Table 5. UHPLC-ToF-MS2-data of yet unidentified or ambiguous hops rich beer types markers 8

Supplementary Table 6. Structural substantiation of wheat grain biomarker masses by means of UHPLC-ToF-MS^2 11

Supplementary Table 7. Instrumental parameters and reagents used for FTICR- and UHPLC-ToF-MS measurements. 12

Figures 13

Supplementary Figure 1. Van Krevelen diagram of sum formulae present in >95 % of all beers 13

Supplementary Figure 2. OPLS-DA-models' loading plots 14

Supplementary Figure 3. OPLS-DA score plots and dedicated van Krevelen diagrams for the grain and color models 15

Supplementary Figure 4. Intensity distribution for the selected wheat grain markers (heatmap) 16

Supplementary Figure 5. UHPLC-ToF-MS chromatogram with EICs of hops rich beer types markers found by FT-ICR-MS. Exemplary EIC of cohulupone and humulinone isomeric compounds. Comparison of the mass traces of identified markers between a hops rich craft beer and a wheat beer sample. UHPLC-ToF-MS EICs of wheat grain marker masses. 18

Supplementary Figure 6. Van Krevelen diagram of eleven possible compositions for m/z 362.05072 (3 ppm) and isotopic fine structure of $[C_{10}H_{13}N_5O_8P]^-$ 19

References 20

Tables

Supplementary Table 1. Overview of the OPLS-DA models (exclusions, predictions) and statistical parameters (R^2Y, Q^2, VC-ANOVA)

model	no. samples	exclusion	Prediction (of model sample set)	R^2Y	Q^2	ANOVA (p-value)
beertype	78	geuze	*Triticum dicoccum* and *Triticum aestivium spelta* used for wheat beers; wit beer (raw wheat); sample 85 (typical wheat beer)	0.96	0.63	1.13 E-23
grain	81	-	Triticum dicoccum, Triticum aestivium spelta and merely wheat starch	0.98	0.73	2.55 E-19

Supplementary Table 2. Overview (beer type, grain used, scores and set type) of the measured samples' characteristics.

sample no.	beer type	grain	model 1		model 2		sample set	country
			score (x)	score (x)	score (y)	score (y)		
01	Wheat	Wheat	17.93	1.33	-16.17	2.24	model	GER
02	Lager	Barley	-10.95	13.23	7.94	16.76	model	GER
03	Lager	Barley	-10.82	13.55	9.17	13.05	model	GER
04	Abbey	Barley	-1.00	1.21	9.48	-13.03	model	BEL
05	Wheat	Wheat	20.29	-2.80	-17.59	-22.83	model	GER
06	Wheat	Wheat	22.54	-0.60	-16.98	-17.74	model	GER
07	Wheat	Wheat	19.49	-0.07	-17.30	-3.92	model	GER
08	Lager	Barley	-10.09	13.34	11.10	-8.80	model	GER
09	Lager	Barley	-10.62	12.38	9.49	11.52	model	GER
10	Lager	Barley	-10.15	8.21	7.57	20.13	model	GER
11	Wheat	Wheat	17.59	4.94	-12.08	16.53	model	GER
12	Wheat	Wheat	19.04	-2.03	-14.82	-1.58	model	GER
13	Lager	Barley	-10.11	8.49	7.31	13.35	model	GER
14	Lager	Barley	-7.39	10.04	6.67	6.94	model	CZE
15	Lager	Barley	-9.69	13.77	10.08	9.40	model	GER
16	Lager	Barley	-9.54	11.57	10.25	10.47	model	GER
17	Abbey	Barley	-1.82	1.36	10.83	-6.38	model	BEL
18	Wheat	Wheat	20.99	-0.55	-17.32	-10.85	model	USA
19	Wheat	Wheat	19.47	-0.66	-14.74	4.4	model	GER
20	Wheat	Wheat	14.76	0.15	-12.2	6.57	model	GER
21	Lager	Barley	-8.25	6.99	9.61	-3.43	model	GER
22	Lager	Barley	-8.36	10.13	10.1	-0.20	model	GER
23	Lager	Barley	-9.36	6.32	10.34	-3.63	model	GER
24	Wheat	Wheat	16.68	-0.82	-14.7	0.10	model	IRL
25	Lager	Barley	-12.5	11.96	10.24	-19.22	model	GER
26	Lager	Barley	-7.42	12.61	9.65	9.30	model	GER
27	Wheat	Wheat	20.85	0.39	-16.41	-14.00	model	GER
28	Lager	Barley	-9.42	14.09	10.43	6.71	model	GER
29	Wheat	Wheat	18.52	2.04	-16.74	-6.81	model	GER
30	Wheat	Wheat	20.01	-0.63	-15.85	-17.76	model	GER
31	Wheat	Wheat	20.90	-0.99	-17.21	-7.81	model	GER
32	Lager	Barley	-9.32	11.58	8.04	7.88	model	BEL
33	Lager	Barley	-7.76	11.80	8.70	4.00	model	GER
34	Lager	Barley	-14.35	11.45	11.43	-18.68	model	GER
35	Wheat	Wheat	20.17	1.93	-17.17	-7.20	model	GER
36	Lager	Barley	-7.23	13.64	9.46	-1.9	model	GER
37	Abbey	Barley	-0.01	0.54	8.33	-19.71	model	GER
38	Lager	Barley	-9.38	12.08	11.13	0.91	model	GER
39	Abbey	Barley	-0.16	1.90	8.18	-1.21	model	GER
40	Lager	Barley	-8.36	13.84	10.46	10.75	model	BEL
41	Wheat	Wheat	21.64	1.50	-17.67	-4.25	model	GER
42	Lager	Barley	-10.08	12.75	11.87	7.49	model	GER
43	Lager	Barley	-10.81	7.98	9.22	11.81	model	BEL
44	Lager	Barley	-12.85	8.46	10.08	-28.41	model	GER
45	Craft	Barley	-9.02	-23.15	8.98	30.98	model	GER
46	Lager	Barley	-10.2	8.54	10.04	0.94	model	GER
47	Wheat	Wheat	20.14	-3.26	-16.69	-5.28	model	GER
48	Craft	Barley	-7.91	-21.61	10.10	12.25	model	GER
49	Wheat	Wheat	20.19	1.04	-17.10	-12.99	model	GER
50	Craft	Barley	-7.59	-19.19	9.03	3.44	model	GER
51	Wheat	Wheat	21.67	1.40	-16.46	-1.82	model	GER
52	Craft	Barley	-8.70	-21.01	9.32	21.08	model	GER
53	Craft	Wheat	-7.84	-19.04	-12.98	30.57	model	BEL
54	Craft	Wheat	-7.44	-23.61	-16.42	30.32	model	LTU
55	Wheat	Wheat	18.79	1.47	-15.61	-12.02	model	GER

56	Craft	Barley	-8.16	-18.04	7.47	8.13	model	GER
57	Abbey	Barley	-1.06	3.02	10.65	-9.46	model	GER
58	Abbey	Barley	-0.33	2.45	11.78	-24.42	model	BEL
59	Geuze	Barley	excl.	excluded	11.46	-27.46	model	GER
60	Lager	Barley	-9.2	15.31	10.71	-0.06	model	GER
61	Lager	Barley	-6.95	12.88	10.69	-10.24	model	GER
62	Lager	Barley	-7.6	10.96	10.08	-9.79	model	GER
63	Lager	Barley	-8.52	12.71	9.04	1.1	model	GER
64	Wheat	Wheat	19.22	1.90	-15.32	8.66	model	BEL
65	Wheat	Wheat	20.31	-1.80	-17.33	-8.44	model	BEL
66	Wheat	Wheat	19.68	0.64	-16.27	1.12	model	GER
67	Craft	Barley	-9.87	-27.03	8.27	5.71	model	GER
68	Lager	Barley	-10.88	7.69	7.80	7.33	model	BEL
69	Lager	Barley	-8.49	8.63	10.41	-4.48	model	NAM
70	Craft	Barley	-10.03	-27.71	7.93	20.05	model	DNK
71	Craft	Barley	-12.03	-21.12	9.26	-6.71	model	GER
72	Craft	Barley	-10.87	-20.19	10.70	-22.94	model	GER
73	Craft	Wheat	-9.76	-24.97	-11.46	26.61	model	GER
74	Craft	Barley	-10.52	-23.65	9.12	22.49	model	GER
75	Craft	Barley	-8.66	-22.2	7.76	-20.98	model	GER
76	Craft	Wheat	-5.50	-19.07	-11.84	15.57	model	GER
77	Abbey	Wheat(starch)[a]	-0.92	3.65	9.27	-11.75	model	GER
78	Craft	Emmer[b]	-10.69	-16.62	5.94	-20.77	model	GER
79	Geuze	Barley	excl.	excluded	14.10	-32.85	model	GER
80	Craft	Wheat	-4.29	-17.34	-15.46	8.86	model	GER
81	excl.	excluded	excl.	excluded	excluded	excluded	model	GER
82	Wheat	Wheat	5.70	-6.08	-14.58	7.15	model	GER
83	Wheat	Wheat	17.07	-0.68	-16.65	-7.00	model	GER
84	Wit	Wheat(raw)[c]	9.18	0.53	-12.73	3.59	model	GER
85	Wheat	Spelt[d]	15.27	0.38	-13.49	-3.76	model	GER
86	Lager	Barley	-2.79	1.07	5.21	-6.72	prediction	CUB
87	Lager	Barley	-2.57	5.82	5.22	-4.98	prediction	CUB
88	Lager	Barley	-6.64	-1.86	6.06	-4.64	prediction	MEX
89	Lager	Barley	-1.71	3.88	2.83	0.84	prediction	MEX
90	Lager	Barley	-3.67	-0.10	4.03	3.09	prediction	CHN
91	Lager	Barley	-6.54	3.26	8.49	-4.32	prediction	PER
92	Lager	Barley	-2.05	0.57	5.53	-6.20	prediction	ARG
93	Lager	Barley	-3.68	-3.77	3.3	-19.06	prediction	PER
94	Lager	Barley	-2.77	0.01	2.91	2.52	prediction	ESP
95	Lager	Barley	-2.70	-1.89	9.35	-14.11	prediction	BRA
96	Craft	Barley	-5.28	-8.41	6.01	5.74	prediction	JPN
97	Wheat	Wheat	5.77	-2.03	-5.43	6.64	prediction	NLD
98	Lager	Barley	-4.91	4.01	7.29	-1.71	prediction	KOR
99	Abbey	Wheat(raw)[c]	6.79	-0.41	-0.71	-5.15	prediction	BEL
100	Abbey	Wheat	-3.63	-9.37	2.98	-12.19	prediction	NLD
101	Abbey	Wheat(starch) [a]	-1.32	-0.54	5.79	-2.10	prediction	BEL
102	Abbey	Wheat(raw)[c]	10.39	-1.13	-6.06	-1.19	prediction	BEL
103	Craft	Barley	-5.56	-12.21	1.98	-6.02	prediction	BEL
104	Lager	Barley	-8.36	-4.28	7.7	9.01	prediction	NLD
105	Lager	Barley	-7.94	0.27	6.96	7.05	prediction	NLD
106	Craft	Barley	-8.00	-15.59	5.26	15.03	prediction	NLD
107	Lager	Barley	-6.36	-6.18	3.45	5.15	prediction	GER
108	Lager	Barley	-4.74	7.55	7.14	-2.31	prediction	SGP
109	Wheat	Wheat	9.61	-6.5	-7.35	-2.52	prediction	NDL
110	Abbey	Wheat(starch) [a]	0.87	0.13	5.83	-10.34	prediction	BEL
111	Craft	Barley	3.05	1.98	3.19	-7.33	prediction	BEL
112	Craft	Barley	-8.09	-14.45	6.13	-8.40	prediction	NDL
113	Abbey	Barley	-2.15	1.93	5.62	-1.56	prediction	NDL
114	Craft	Wheat	3.10	-6.70	0.35	-18.18	prediction	GER
115	Abbey	Barley	0.35	2.77	6.91	-8.92	prediction	BEL
116	Craft	Barley	-3.87	-12.17	5.19	-15.78	prediction	BEL

117	Lager	Barley	-8.86	-5.33	5.58	-5.06	prediction	GER
118	Craft	Wheat	-1.41	-6.82	2.02	-13.26	prediction	GER
119	Abbey	Barley	-6.81	-12.55	6.47	2.71	prediction	BEL
120	Craft	Wheat(raw)[c]	3.57	-5.55	-1.41	3.94	prediction	BEL

[a] Triticum aestivium starch only
[b] Triticum dicoccum
[c] Triticum aestivium not malted (typical for wit beers)
[d] Triticum aestivium subsp. spelta

Supplementary Table 3. Tentative annotations of markers for rich hopped beers on basis of exact masses.

$m/z_{measured}$	m/z $[M\text{-}H]^-_{theor.}$	error [ppm]	sum for-	annotation	literature
263.12890	263.12888	0.05	$C_{15}H_{20}O_4$	phenylphlorisobutyrophenone, hulupinic acid	1,2
265.14453	265.14453	0.01	$C_{15}H_{22}O_4$	humulinic acid, adhumulinic acid	1
277.14455	277.14453	0.06	$C_{16}H_{22}O_4$	phenylphlorisoalerophenone	2
281.13946	281.13945	0.02	$C_{15}H_{22}O_5$	oxyhumulinic acid	3
317.17582	317.17583	0.05	$C_{19}H_{26}O_4$	cohulupone	1
331.19146	331.19148	0.08	$C_{20}H_{28}O_4$	deoxycohumulone, hulupone, adhulupone	1
345.20711	345.20713	0.06	$C_{21}H_{30}O_4$	deoxyhumulone, deoxyadhumulone	1
347.18649	347.18640	0.25	$C_{20}H_{28}O_5$	(allo)cohumulone, (allo)iso-cohumulone, (iso)-tricycloco-humene	1
349.20203	349.20205	0.05	$C_{20}H_{30}O_5$	dihydrocohumulone	3
361.20214	361.20204	0.27	$C_{21}H_{30}O_5$	humulone, (allo)(iso)-(ad)humulone, (iso)-(ad)tricyclohu-mene	1
363.18134	363.18131	0.08	$C_{20}H_{28}O_6$	(iso)cohumulinone, hydroxyl-alloisocohumulone, scorpi-ocohumol	1
363.21771	363.21770	0.03	$C_{21}H_{32}O_5$	dihydrohumulone	3
365.19700	365.19696	0.10	$C_{20}H_{30}O_6$	cohumol, tricyclocohumol, (epi)tetracyclocohumol, hy-droxyl(iso)cohumulon	1,4,5
377.19701	377.19696	0.14	$C_{21}H_{30}O_6$	(iso)humulinone, adhumulinone, hydroxyl-alloiso(ad)hu-mulone, scorpiohumol	1
379.21264	379.21261	0.08	$C_{21}H_{32}O_6$	humol, tricyclohumol, tetracyclohumol	1
381.19187	381.19188	0.01	$C_{20}H_{30}O_7$	hydroxyl-alloisocohumulonhydroxid	4
393.19196	393.19188	0.22	$C_{21}H_{30}O_7$	allosiohumulonhydroperxid	4
431.24395	431.24391	0.08	$C_{25}H_{36}O_6$	colupdox, hydroxyperoxytricyclocolupon	6,7
433.25958	433.25956	0.05	$C_{25}H_{38}O_6$	hydroperoxitricyclolupone	1
445.25960	445.25956	0.09	$C_{26}H_{38}O_6$	lupdox	6

Supplementary Table 4. Structural identification of hops rich beer type marker masses by means of UHPLC-ToF-MS^2. Level of identification 2.

$m/z_{measured}$	m/z [M-H]$^-$ theor.	error [ppm]	sum formula	Rt[a] [min]	compound	MS^2 fragments [m/z (rel. intensity)]	lit.[b]
251.1289	251.12888	-0.08	$C_{14}H_{20}O_4$	4.8	cohumulinic acid	71(23), 113(34), **141(100)**, 165(72),	5
263.1290	263.12880	-0.76	$C_{15}H_{20}O_4$	4.6	hulupinic acid	126(21), 151(96), 165(19), 179(37),	7
317.1760	317.17583	0.54	$C_{19}H_{26}O_4$	5.3	cohulupone	180(28), 184(23), **205(100)**, 220(35), 233(69), 248(25)	8
347.1863	347.18640	0.29	$C_{20}H_{28}O_5$	6.0	iso-cohumulone	**181(100)**, 207(11), 209(35), 233(35), 235(10), 251(62), 278(8), 329(4)	9
347.1858	347.18640	1.73	$C_{20}H_{28}O_5$	6.5	cohumulone	207(35), **235(100)**, 278(84)	9
349.2024	349.20205	1.00	$C_{20}H_{30}O_5$	6.6	dihydrocohumu-lone	207(11), 209(7), 235(30), 237(24), 278(21), **280(100)**	9[c]
361.2032	361.20204	3.21	$C_{21}H_{30}O_5$	6.1	iso-(ad)humu-lone	**195(100)**, 221(20), 223(54), 247(59), 265(23), 343(6)	9
361.2033	361.20204	3.49	$C_{21}H_{30}O_5$	6.6	(ad)humulone	221(36), 249(90), **292(100)**	9
365.1977	365.19696	2.03	$C_{20}H_{30}O_6$	3.8	tricyclocohumol	165(89), **183(100)**, 245(8), 277(7), 289(6), 303(9), 347(86)	10
365.1976	365.19696	-1.75	$C_{20}H_{30}O_6$	4.2	hydroxyl(iso)co-humulon	181(38), 193(64), **251(100)**, 269(71), 296(21)	4
365.1972	365.19696	0.66	$C_{20}H_{30}O_6$	4.8	tetracyclohumol	165(55), **193(100)**, 289(21), 307(82), 347(3)	10
379.2131	379.21261	1.29	$C_{21}H_{32}O_6$	4.2	tricyclohumol	179(73), **197(100)**, 277(6), 303(8), 317(6), 361(80)	10

[a] retention time

[b] literature

[c] The literature data refers to the dedicated non-hydrated compound. Level of identification 3.

Supplementary Table 5. UHPLC-ToF-MS²-data of ambiguous marker substances for rich hopped beer. The compound class is putatively characterized as hops terpeno-phenolics (hops bitter acids and their derivatives) (level of identification 3[b]). The five highest fragments are shown.

*m/z*measured	*m/z* [M-H]⁻ theor.	error [ppm]	sum formula	Rt[a] [min]	MS² fragments [*m/z* (rel. intensity)]
251.1286	251.12888	1.11	$C_{14}H_{20}O_4$	4.4	**69(100)**, 81(6), 98(11), 189(6), 251(6)
251.1289	251.12888	-0.08	$C_{14}H_{20}O_4$	4.8	71(23), 113(34), **141(100)**, 165(72), 233(40)
251.1289	251.12888	-0.08	$C_{14}H_{20}O_4$	5.8	65(85), 111(28). **152(100)**, 180(11), 184(62)
263.1288	263.12880	0.00	$C_{15}H_{20}O_4$	5.8	97(40), 163(33), 177(35), **192(100)**, 205(90)
263.1291	263.12880	-1.14	$C_{15}H_{20}O_4$	6.0	82(14), 124(18), **166(100)**, 179(22), 198(46)
265.1444	265.14453	0.49	$C_{15}H_{22}O_4$	5.1	69(13), 127(9), **155(100)**, 179(18), 247(9)
265.1448	265.14453	0.33	$C_{15}H_{22}O_4$	5.7	79(11), **97(100)**, 177(2), 205(3), 265(20),
277.1451	277.14453	-2.06	$C_{16}H_{22}O_4$	4.4	137(13), **165(100)**, 175(27), 190(20), 208(29)
279.1239	279.12380	-0.36	$C_{15}H_{20}O_5$	3.5	139(9), **167(100)**, 181(13), 195(9), 210(18)
279.1237	279.12380	0.36	$C_{15}H_{20}O_5$	3.8	**165(100)**, 167(18), 181(13),183(9), 210(9)
279.1237	279.12380	0.36	$C_{15}H_{20}O_5$	5.1	139(13), 165(9), **167(100)**, 210(18), 249(9)
279.1238	279.12380	0.00	$C_{15}H_{20}O_5$	5.3	65(18), 133(9), 151(9), 207(13), **235(100)**
279.1236	279.12380	0.72	$C_{15}H_{20}O_5$	5.9	65(18), 151(13), **235(100)**, 261(9), 279(9)
281.1382	281.13945	4.45	$C_{15}H_{22}O_5$	3.9	73(9), 139(9), 155(18), 165(9), **195(100)**
281.1397	281.13945	-0.89	$C_{15}H_{22}O_5$	4.0	66(9), 101(9), 155(9), 177(9), **195(100)**
293.1393	293.13945	0.51	$C_{16}H_{22}O_5$	4.1	153(18), **179(100)**, 195(9), 197(13), 224(9)
295.1552	295.15510	-0.34	$C_{16}H_{24}O_5$	3.6	153(71), 167(33), 181(25), **226(100)**, 277(30)
305.1395	305.13945	-0.16	$C_{17}H_{22}O_5$	4.3	165(12), 193(14), 231(11), 243(27), **305(100)**
305.1394	305.13945	0.16	$C_{17}H_{22}O_5$	5.2	149(14), **193(100)**, 248(11), 288(7), 303(14)
317.1395	317.13945	-0.16	$C_{18}H_{22}O_5$	4.2	147(90), 161(89), 179(57), 192(71), **233(100)**
317.1395	317.13945	-0.16	$C_{18}H_{22}O_5$	4.3	**180(100)**, 192(97), 220(71), 261(43), 289(66)
317.1395	317.13945	-0.16	$C_{18}H_{22}O_5$	5.4	205(43), 262(58), 274(30),302(30), **317(100)**
319.1551	319.15510	0.00	$C_{18}H_{24}O_5$	4.6	193(10), 217(8), 235(8), 257(12), **319(100)**
319.1551	319.15510	0.00	$C_{18}H_{24}O_5$	4.8	165(9), 193(13), 245(6), 257(14), **319(100)**
319.1551	319.15510	0.00	$C_{18}H_{24}O_5$	5.6	165(17), 179(50), 195(17), **207(100)**, 250(55)
329.1758	329.17583	0.09	$C_{20}H_{26}O_4$	5.2	**167(100)**, 195(41), 201(23), 219(43), 242(29)
331.1917	331.19148	-0.66	$C_{20}H_{28}O_4$	5.5	166(92), 191(44), 194(49), **219(100)**, 247(68)
331.1915	331.19148	-0.06	$C_{20}H_{28}O_4$	5.6	166(78), **205(100)**, 219(77), 234(40), 247(76)
333.1710	333.17075	-0.75	$C_{19}H_{26}O_5$	3.8	205(25), **221(100)**, 236(27), 247(42), 249(90)
333.1710	333.17075	-0.75	$C_{19}H_{26}O_5$	3.9	152(58), 181(80), 205(69), 233(70), **247(100)**
333.1712	333.17075	-1.35	$C_{19}H_{26}O_5$	4.9	169(22), 181(10), 221(11), **237(100)**, 335(9)
333.1708	333.17075	-0.15	$C_{19}H_{26}O_5$	5.1	151(8), **167(100)**, 193(13), 195(35), 219(34)
333.1709	333.17075	-0.45	$C_{19}H_{26}O_5$	5.2	163(19), **167(100)**, 193(9), 195(39), 219(34)
333.1710	333.17075	-0.75	$C_{19}H_{26}O_5$	6.0	179(23), 193(59), 209(24), **221(100)**, 264(81)
335.1497	335.15001	0.92	$C_{18}H_{24}O_6$	3.3	165(10), 245(7), 251(6), 317(6), **335(100)**
335.1499	335.15001	0.33	$C_{18}H_{24}O_6$	3.5	209(4), 245(5), 251(4), 273(7), **335(100)**
335.1496	335.15001	1.22	$C_{18}H_{24}O_6$	4.0	181(62), 205(79), 233(80), **247(100)**, 291(67)
335.1496	335.15001	1.22	$C_{18}H_{24}O_6$	4.5	196(28), 221(32), **247(100)**, 265(26), 335(29)
335.1496	335.15001	1.22	$C_{18}H_{24}O_6$	6.8	182(17), **195(100)**, 238(22), 247(29), 263(23)
335.1858	335.18640	1.79	$C_{19}H_{28}O_5$	4.8	59(34), 85(46), 203(87), 219(39), **263(100)**
335.1863	335.18640	0.30	$C_{19}H_{28}O_5$	5.0	169(22), 204(21), **237(100)**, 247(14), 335(67)
335.1864	335.18640	0.00	$C_{19}H_{28}O_5$	5.1	**181(100)**, 231(47), 249(59), 259(51), 265(84)
335.1862	335.18640	0.60	$C_{19}H_{28}O_5$	5.2	85(44), 203(69), 235(34), **263(100)**, 273(41)
335.1863	335.18640	0.30	$C_{19}H_{28}O_5$	5.3	115(44), 153(23), **167(100)**, 195(39), 219(35)
335.1858	335.18640	1.79	$C_{19}H_{28}O_5$	5.4	166(44), 187(43), 191(54), **231(100)**, 235(31)

335.1862	335.18640	0.60	$C_{19}H_{28}O_5$	5.6	166(83), 191(47), 194(47), **219(100)**, 247(76)
343.1914	343.19148	0.23	$C_{21}H_{28}O_4$	5.0	190(71), 205(65), **231(100)**, 259(73), 343(50)
343.1915	343.19148	-0.06	$C_{21}H_{28}O_4$	5.5	**181(100)**, 187(56), 191(99), 235(64), 242(52)
343.1918	343.19148	-0.93	$C_{21}H_{28}O_4$	6.0	**163(100)**, 180(99), 247(34), 249(73), 259(36)
345.2073	345.20713	-0.49	$C_{21}H_{30}O_4$	5.6	167(73), 179(65), 191(80), **235(100)**, 249(40)
347.1495	347.15001	1.47	$C_{19}H_{24}O_6$	4.9	164(81), 191(53), **207(100)**, 234(48), 305(44)
347.1863	347.18640	0.29	$C_{20}H_{28}O_5$	4.1	219(29), 221(25), 235(26), 261(37), **263(100)**
347.1861	347.18640	0.86	$C_{20}H_{28}O_5$	5.3	165(40), 167(49), 181(38), 223(39), **251(100)**
349.1657	349.16566	-0.11	$C_{19}H_{26}O_6$	4.0	167(20), 201(32), 235(51), **263(100)**, 347(24)
349.2020	349.20205	0.14	$C_{20}H_{30}O_5$	4.8	164(34), 207(77), **221(100)**, 253(92), 305(75)
351.1813	351.18131	0.03	$C_{19}H_{28}O_6$	4.6	166(17), **193(100)**, 239(11), 263(32), 351(14)
351.1810	351.18131	0.88	$C_{19}H_{28}O_6$	4.9	181(7), 195(9), 209(26), 223(58), 292(100)
351.1813	351.18131	0.03	$C_{19}H_{28}O_6$	5.0	177(63), **191(100)**, 207(76), 303(65), 349(79)
359.1868	359.18640	-1.11	$C_{21}H_{28}O_5$	5.3	194(20), 222(17), 233(9), 292(11), **359(100)**
359.1866	359.18640	-0.56	$C_{21}H_{28}O_5$	5.5	205(94), 221(32), 249(49), **263(100)**, 359(28)
359.1866	359.18640	-0.56	$C_{21}H_{28}O_5$	5.6	183(71), 189(75), 195(61), 295(60), **359(100)**
359.1867	359.18640	-0.84	$C_{21}H_{28}O_5$	5.8	167(63), 179(42), 191(81), 195(75), **235(100)**
359.1867	359.18640	-0.84	$C_{21}H_{28}O_5$	5.9	179(30), 191(28), 195(35), 235(38), **263(100)**
359.1867	359.18640	-0.84	$C_{21}H_{28}O_5$	6.1	99(23), **195(100)**, 223(49), 247(52), 265(23)
359.1865	359.18640	-0.28	$C_{21}H_{28}O_5$	6.2	**195(100)**, 223(35), 247(35), 263(25), 265(15)
363.1815	363.18131	-0.52	$C_{20}H_{28}O_6$	3.8	231(5), 251(3), 345(4), **363(100)**
363.1815	363.18131	-0.52	$C_{20}H_{28}O_6$	3.9	165(5), 183(6), 301(3), 347(6), **363(100)**
363.1817	363.18131	-1.07	$C_{20}H_{28}O_6$	4.3	164(39), **181(100)**, 195(39), 233(34), 235(25)
363.1818	363.18131	-1.35	$C_{20}H_{28}O_6$	4.4	167(7), 206(7), 209(15), 225(7), **249(100)**
363.1814	363.18131	-0.25	$C_{20}H_{28}O_6$	4.5	**181(100)**, 195(48), 233(34), 249(44), 253(32)
363.1816	363.18131	-0.80	$C_{20}H_{28}O_6$	4.7	85(27), 139(18), 167(41), 209(88), **249(100)**
365.1981	365.19696	-3.12	$C_{20}H_{30}O_6$	5.2	195(6), 209(9), 223(28), 237(55). **296(100)**
375.1815	375.18131	-1.70	$C_{21}H_{28}O_6$	4.3	64(65), **80(100)**, 191(86), 277(22), 295(21)
377.1976	377.19696	-1.70	$C_{21}H_{30}O_6$	4.0	245(4), 251(3), 293(2), 359(4), **377(100)**
377.1977	377.19696	-1.96	$C_{21}H_{30}O_6$	4.1	245(2). 251(3), 293(3), 315(2), **377(100)**
377.1974	377.19696	-1.17	$C_{21}H_{30}O_6$	4.2	80(2), 245(2), 251(2), 315(2), **377(100)**
377.1974	377.19696	-1.17	$C_{21}H_{30}O_6$	4.5	152(18), **195(100)**, 207(24), 265(21), 283(17)
377.1976	377.19696	-1.7	$C_{21}H_{30}O_6$	4.7	195(8), 220(8), 223(14), 239(8), **263(100)**
379.2132	379.21261	-1.56	$C_{21}H_{32}O_6$	4.1	179(2), 245(3), 293(4), 315(3), **377(100)**
379.2147	379.21261	-5.51	$C_{21}H_{32}O_6$	5.5	223(7), 237(25), 251(52), 254(11), **310(100)**
381.1913	381.19188	1.52	$C_{20}H_{30}O_7$	2.9	245(3), 273(3), 275(3), **337(100)**, 381(63)
381.1921	381.19188	-0.58	$C_{20}H_{30}O_7$	3.1	**181(100)**, 193(24), 361(28), 363(20), 379(18)
381.1920	381.19188	-0.31	$C_{20}H_{30}O_7$	3.3	**181(100)**, 193(18), 233(9), 363(13), 281(70)
381.1913	381.19188	1.52	$C_{20}H_{30}O_7$	3.5	181(85), **193(100)**, 249(73), 305(6)), 379(84)
381.1922	381.19188	-0.84	$C_{20}H_{30}O_7$	3.7	181(84), 193(40), 265(30), 305(59), **379(100)**
383.2072	383.20753	0.86	$C_{20}H_{32}O_7$	3.7	165(60), 183(34), 267(30), **325(100)**, 381(49)
389.1969	389.19696	0.15	$C_{22}H_{30}O_6$	5.6	249(19), 263(26), 265(11), 277(60), **320(100)**
389.1964	389.19696	1.44	$C_{22}H_{30}O_6$	5.8	250(50), 261(44), 263(39), **306(100)**, 389(41)
389.1968	389.19696	0.41	$C_{22}H_{30}O_6$	6.1	235(17), **278(100)**, 285(13), 301(42), 329(72)
389.1968	389.19696	0.41	$C_{22}H_{30}O_6$	6.6	247(56), **263(100)**, 277(24), 290(28), 302(40)
391.2130	391.21261	-1.00	$C_{22}H_{32}O_6$	4.9	153(73), 205(82), **225(100)**, 253(48), 263(40)
391.2123	391.21261	0.79	$C_{22}H_{32}O_6$	5.1	209(47), 224(34), **238(100)**, 277(64), 324(86)
391.2126	391.21261	0.03	$C_{22}H_{32}O_6$	5.3	165(21), 237(52), 248(27), **277(100)**, 317(31)
391.2119	391.21261	1.81	$C_{22}H_{32}O_6$	6.7	179(15), 195(18), 237(36), 253(13), **277(100)**

393.2278	393.22826	1.17	$C_{22}H_{34}O_6$	6.2	181(22), 223(15), 233(11), 237(39), **324(100)**
401.2339	401.23335	-1.37	$C_{24}H_{34}O_5$	5.7	221(16), 259(87), 271(42), **289(100)**, 329(12)
403.2120	403.21261	1.51	$C_{23}H_{32}O_6$	5.2	**64(100)**, 80(66), 167(18), 219(11), 237(21)
403.2158	403.21261	-2.95	$C_{23}H_{32}O_6$	5.9	222(18), 235(11). **247(100)**, 250(928), 275(9)
405.1923	405.19188	-1.04	$C_{22}H_{30}O_7$	8.3	62(12), 147(3), 157(2), 263(3), **337(100)**
407.2074	407.20753	0.32	$C_{22}H_{32}O_7$	4.1	179(12), 183(13), **251(100)**, 263(26), 273(16)
407.2074	407.20753	0.32	$C_{22}H_{32}O_7$	4.2	183(18), **263(100)**, 263(24), 277(33), 409(24)
407.2074	407.20753	0.32	$C_{22}H_{32}O_7$	4.3	163(20), 181(54), **221(100)**, 251(21), 263(22)
419.2072	419.20753	0.79	$C_{23}H_{32}O_7$	5.4	191(7), 247(20), 278(7), 291(6), **291(100)**, 350(12)
419.2076	419.20753	-0.17	$C_{23}H_{32}O_7$	5.5	192(22), 219(29), 235(18), 247(71), **306(100)**
431.2431	431.24391	1.88	$C_{25}H_{36}O_6$	5.1	259(18), 317(23), 329(20), **387(100)**, 431(26)
431.2437	431.24391	0.49	$C_{25}H_{36}O_6$	5.2	287(98), 303(65), 314(33), **331(100)**, 431(34)
431.2439	431.24391	0.02	$C_{25}H_{36}O_6$	6.6	265(61), 278(75), **292(100)**, 329(62), 343(85)
433.2593	433.25956	0.6	$C_{25}H_{38}O_6$	5.3	277(80), **301(100)**, 321(47), 389(35), 431(32)
433.2598	433.25956	-0.55	$C_{25}H_{38}O_6$	5.6	207(17), 280(22), 289(18), **305(100)**, 433(66)
445.2595	445.25956	0.13	$C_{26}H_{38}O_6$	5.5	271(36), 301(38), 310(36), **317(100)**, 445(60)
445.2595	445.25956	0.13	$C_{26}H_{38}O_6$	6.8	265(22), 292(92), 299(19), 315(64), **343(100)**
447.2392	447.23883	-0.83	$C_{25}H_{36}O_7$	4.1	267(5), 329(6), 361(9), 377(11), **447(100)**
447.2392	447.23883	-0.83	$C_{25}H_{36}O_7$	4.9	209(23), 239(12), 265(5), 429(7), **447(100)**
447.2386	447.23883	0.51	$C_{25}H_{36}O_7$	5.3	235(31), 247(83), 265(26), 343(32), **351(100)**
457.2236	457.22318	-0.92	$C_{26}H_{34}O_7$	5.0	181(80), 209(65), **249(100)**, 277(39), 329(29)
457.2237	457.22318	-1.14	$C_{26}H_{34}O_7$	5.3	**233(100)**, 249(48), 251(46), 278(80), 329(92)

[a] retention time

[b] The level of identification 3 is characterized by the putative assignment of the chemical class terpeno-phenolics. It is based on the same behavior upon statistical evaluation of metabolome data, a chemical and biochemical connectivity to known compounds through defined corresponding mass transitions, similar physiochemical properties with regard to retention on reversed-phase chromatography and spectral similarity through matching tandem-mass spectrometric fragments.

Supplementary Table 6. Structural identification of wheat grain biomarker masses by means of UPLC-ToF-MS^2. Level of identification 2.

*m/z*measured	*m/z* [M-H]$^-$ theor.	error [ppm]	sum formula	Rt[a] [min]	compound	MS^2 fragments [*m/z* (rel. intensity)]	Collision energy	lit.[b]
326.0880	326.0881	0.4	$C_{14}H_{17}NO_8$	2.6	HBOA-Hexoside	108(12), 118(4), 136(10), 164(100), 326(1)	30 eV	[11,12]
342.0835	342.0830	1.3	$C_{14}H_{17}NO_9$	1.7	**DHBOA**/DIBOA-Hex.[c]	124(17), 134(7), 152(49), 162(15), 180(100),	35 eV	[12,13]
342.0835	342.0835	0.0	$C_{14}H_{17}NO_9$	2.6	DHBOA/**DIBOA**-Hex.[c]	134(9), 162(31), 175 (20), 180(30), 342(100)	10 eV	[11]
356.0988	356.0987	0.2	$C_{15}H_{19}NO_9$	2.8	HMBOA-Hexose	138(9), 148(7), 166(34), 179(9), 194(100), 356(1)	30 eV	[12,13]
436.0558	436.0554	0.6	$C_{15}H_{19}NO_{12}S$	2.5	HMBOA-Hexosesulfate	194(65), 356(96), 436(100)	20 eV	[12,13d]
504.1357	504.1359	0.4	$C_{20}H_{27}NO_{14}$	1.9	**DHBOA**/DIBOA-Dihexoside[c]	162(19), 180(100), 342(15), 504(1)	35 eV	[13]
504.1352	504.1359	1.4	$C_{20}H_{27}NO_{14}$	2.6	DHBOA/**DIBOA**-Dihexoside[c]	134(7), 162(9), 175(100), 504(1)	35 eV	[13]
518.1514	518.1515	0.3	$C_{21}H_{29}NO_{14}$	2.7	HMBOA-Dihexoside	166(11), 194(100)	35 eV	[13]

[a] retention time
[b] literature
[c] differentiation between DHBOA and DIBOA can’t be accomplished with (LC)-MS^2-data only.
[d] the literature data refers to the dedicated de-sulfated compound. Level of identification 3.

Supplementary Table 7. Instrumental parameters and reagents used for FTICR- and UHPLC-ToF-MS measurements

reagent	source
methanol (MeOH)	FLUKA, Sigma-Aldrich (LC-MS grade, CHROMASOLV, St Louis, MO, USA)
acetonitrile (ACN)	FLUKA, Sigma-Aldrich (LC-MS grade, CHROMASOLV, St Louis, MO, USA)
acetic acid	Biosolve (Valkenswaard, NL)
ultrapure water	Milli-Q Integral Water Purification System (Millipore, Billerica, MA, USA)
L-arginine	Sigma-Aldrich (reagent grade >98%, St Louis, MO, USA)
ESI-L Low Concentration Tuning Mix	Agilent (Santa Clara, CA, United States of America)
**FTICR-MS**	**value**
sample preparation	degassing by ultrasonification (10 °C, 5min.); dilution 1:500 in methanol (v:v); separation of precipitated proteins by centrifugation (10,000 rmp, 3min.)
direct injection flowrate	120 $\mu L.h^{-1}$.
ESI capillary voltage	3600 V
time domain	4 mega words
accumulation time	0.25 ms
mass range	*m/z* 120 to 1000
accumulated scans	400
Measurement time	10 min.
external calibration	clusters of arginine (5 $mg.L^{-1}$ in methanol)
internal calibration	in-house calibration list containing 2000 sum formulae, which are highly abundant in beers
**UHPLC-ToF-MS**	**value**
sample preparation	degassing by ultrasonification (10 °C, 5min.); dilution 1:4 in methanol (v:v); separation of precipitated proteins by centrifugation (10,000 rmp, 3min.); evaporation of the supernatant and dissolving in acetonitrile:water (20:80; v:v)
column	RP (C18: 1.7 µm, 2.1 x 100 mm, Acquity^{TM} UPLC BEH^{TM})
flow rate	400 $\mu L\ min^{-1}$
column temperature	40 °C
injection volume	5 µL (partial loop)
gradient profile	95 % A (0.1 % formic acid in water) and 5 % B (0.1 % formic acid in acetonitrile) for 1 min; decreasing to 0.5 % A in 5 min; held for 4 min.
measurement mode	Data dependent analysis with pre-built preference list (based on FTICR data)
measurement time	10 min.
internal calibration	ESI-L Low Concentration Tuning Mix
external calibration	ESI-L Low Concentration Tuning Mix (1:4 diluted in 75% acetonitrile) in the first 0.3 min of each LC-MS run; introduced by a switching valve.
ESI ionization mode	negative
nitrogen flowrate	10 $L\ min^{-1}$
dry heater	200°C
nebulizer pressure	2.0 bar
capillary voltage	4500 V
endplate offset	500 V
MS^2 fragmentation parameters	MRM and data dependent mode; collision energy10 eV to 35 eV

Figures

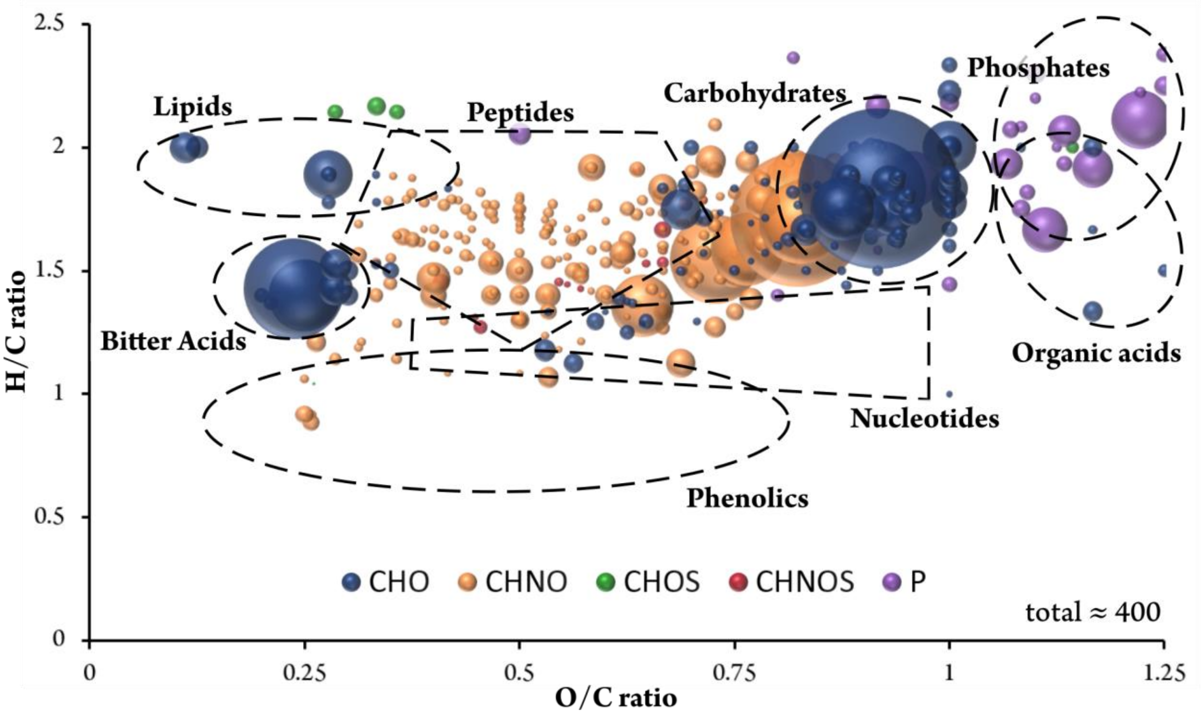

Supplementary Figure 1. Van Krevelen diagram (H/C vs O/C) of the annotated sum formulae appearing in more than 95 % of all beer samples. Areas specific for certain compound classes are marked with dotted lines. Color code of the van Krevelen diagrams: CHO blue; CHNO orange; CHOS green; CHNOS red; P violet; Cl light violet. The bubble size indicate the mean relative intensities of corresponding peaks in the spectra.

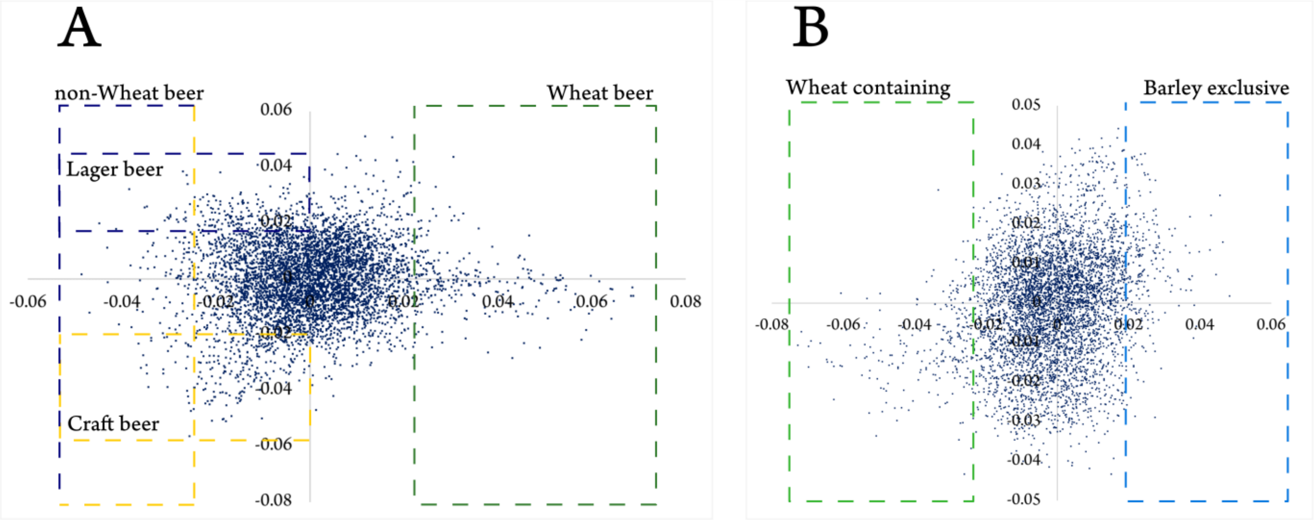

Supplementary Figure 2. OPLS-DA loading plots for the beer type and grain observations. The loadings of the model differentiating the beer types are shown in **(A)**, the loadings for the differentiation of beer brewed with wheat or exclusively barley are shown in **(B)**. The 95th percentile of the different classes' marker substances are marked by colored areas**.**

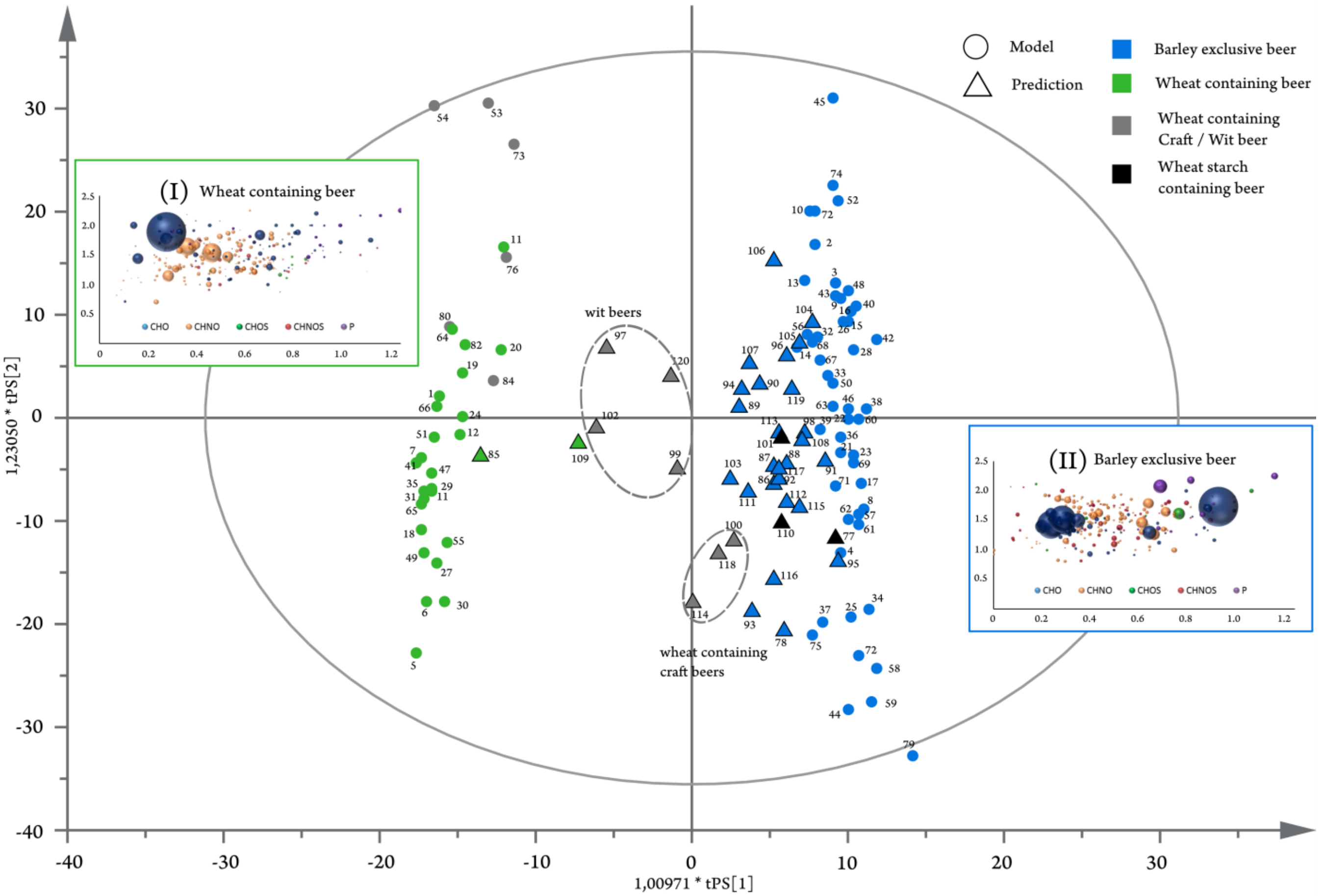

Supplementary Figure 3. OPLS-DA model's score plot for the wheat containing (green) and beers brewed with barley exclusively (blue) observation. The model sample set is depicted as circles, the prediction set is depicted as triangles. Different grain types are indicated by different colors. The score plots are surrounded by the observations' van Krevelen diagrams. Color code and bubble size of the van Krevelen diagrams see Figure S2. Samples included in the model calculation are depicted as circles, whereas predicted samples are represented as triangles.

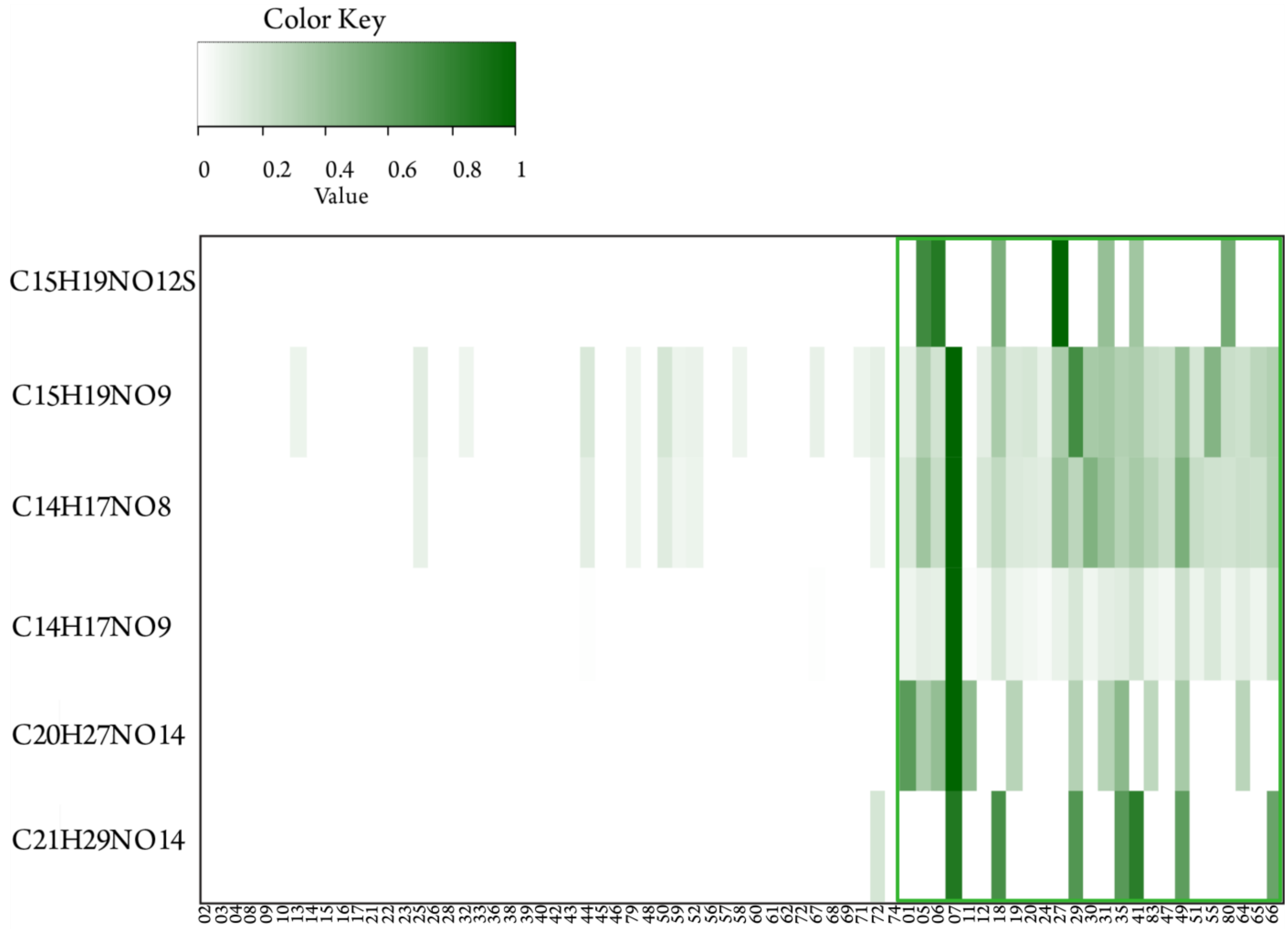

Supplementary Figure 4. Intensity distribution for the wheat grain markers. $C_{14}H_{17}NO_8$ (HBOA-hex.), $C_{14}H_{17}NO_9$ (DHBOA/DIBOA-hex.), $C_{15}H_{19}NO_9$ (HMBOA-hex.), $C_{15}H_{19}NO_{12}S$ (HMBOA-hex.sulfate), $C_{20}H_{27}NO_{14}$ (DHBOA/DIBOA-dihex.), $C_{21}H_{29}NO_{14}$ (HMBOA-dihex.) are depicted. The maximum intensity for every peak is set to 100%. Beers brewed with wheat grain are marked. Trace amounts of the markers' corresponding masses in exclusively barley containing beers might occur due to isomeric compounds.

A

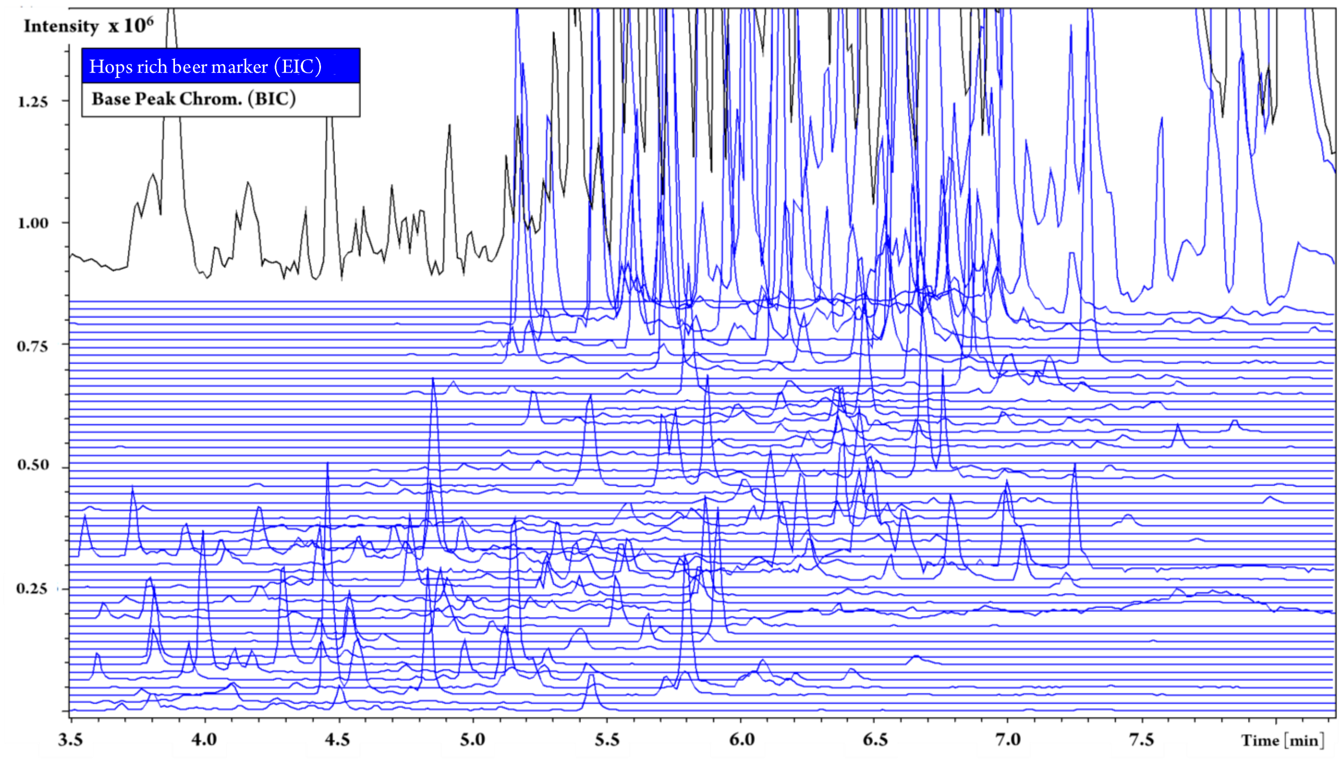

B

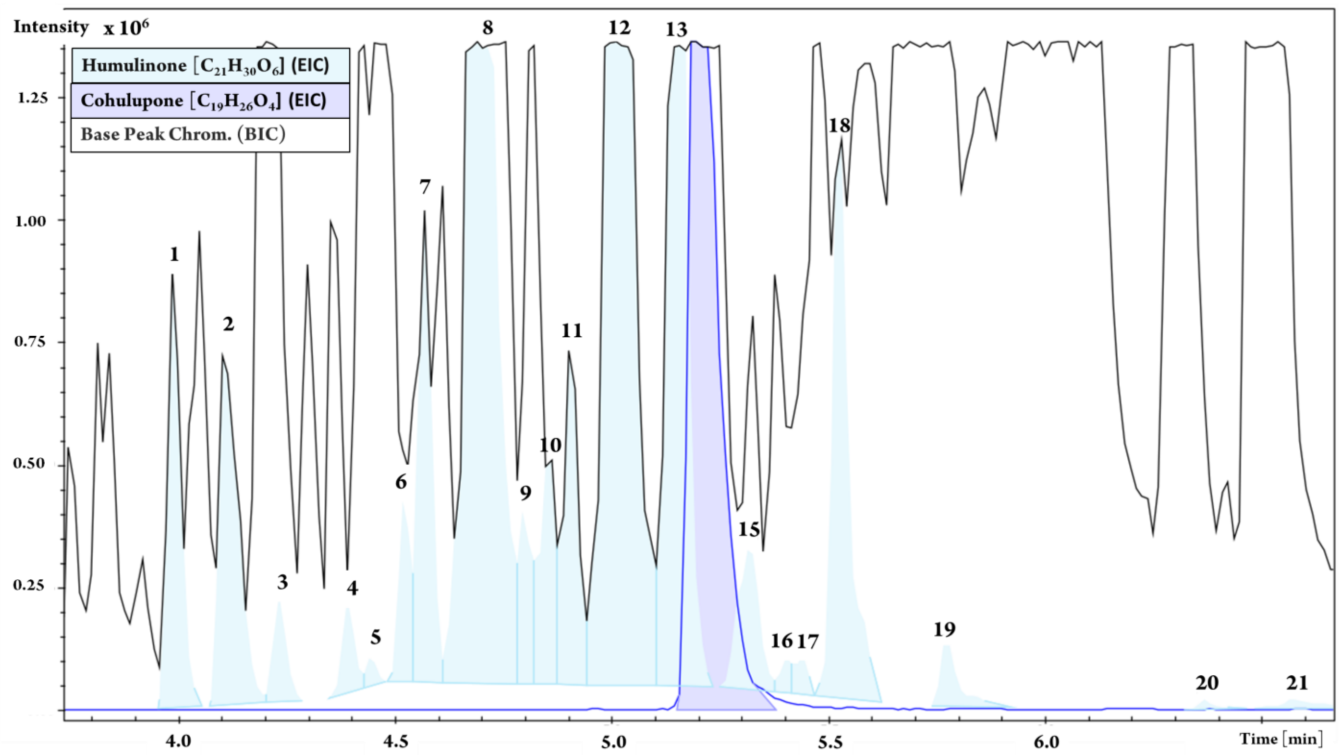

Supplementary Figure 5. continued.

C

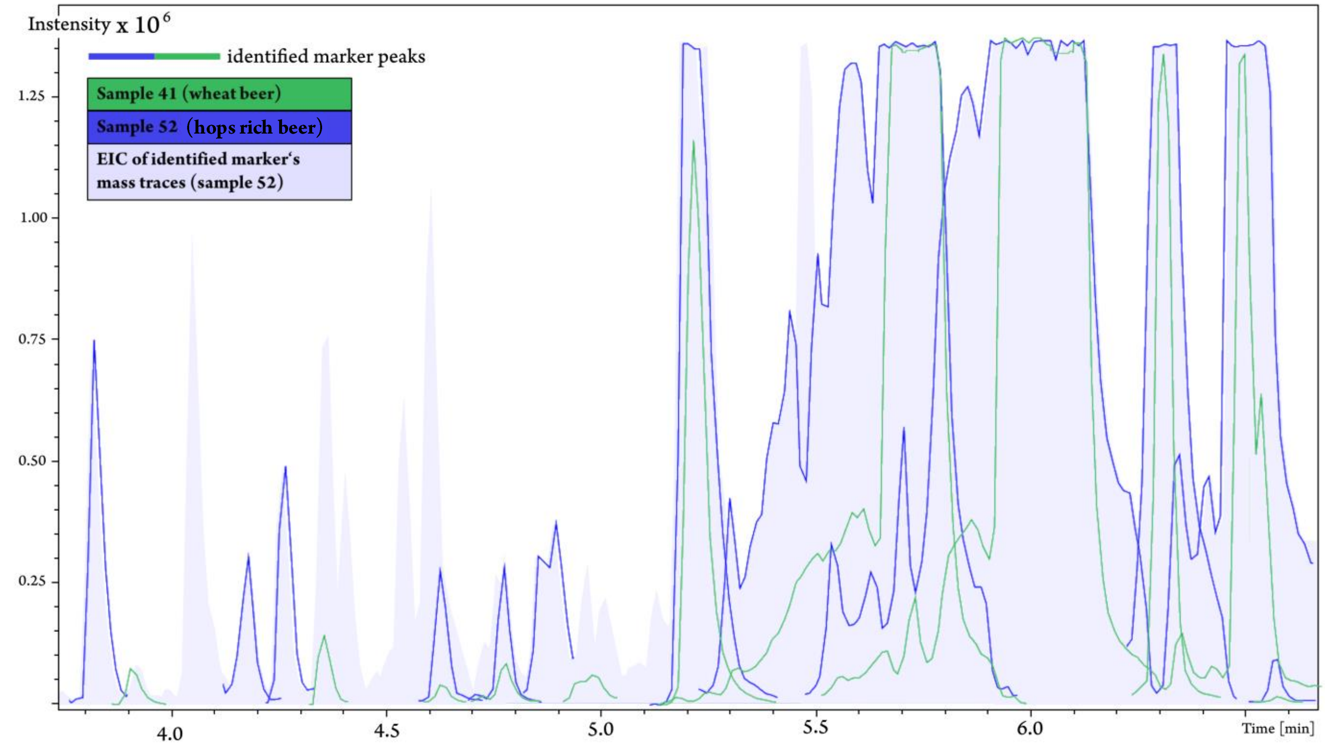

D

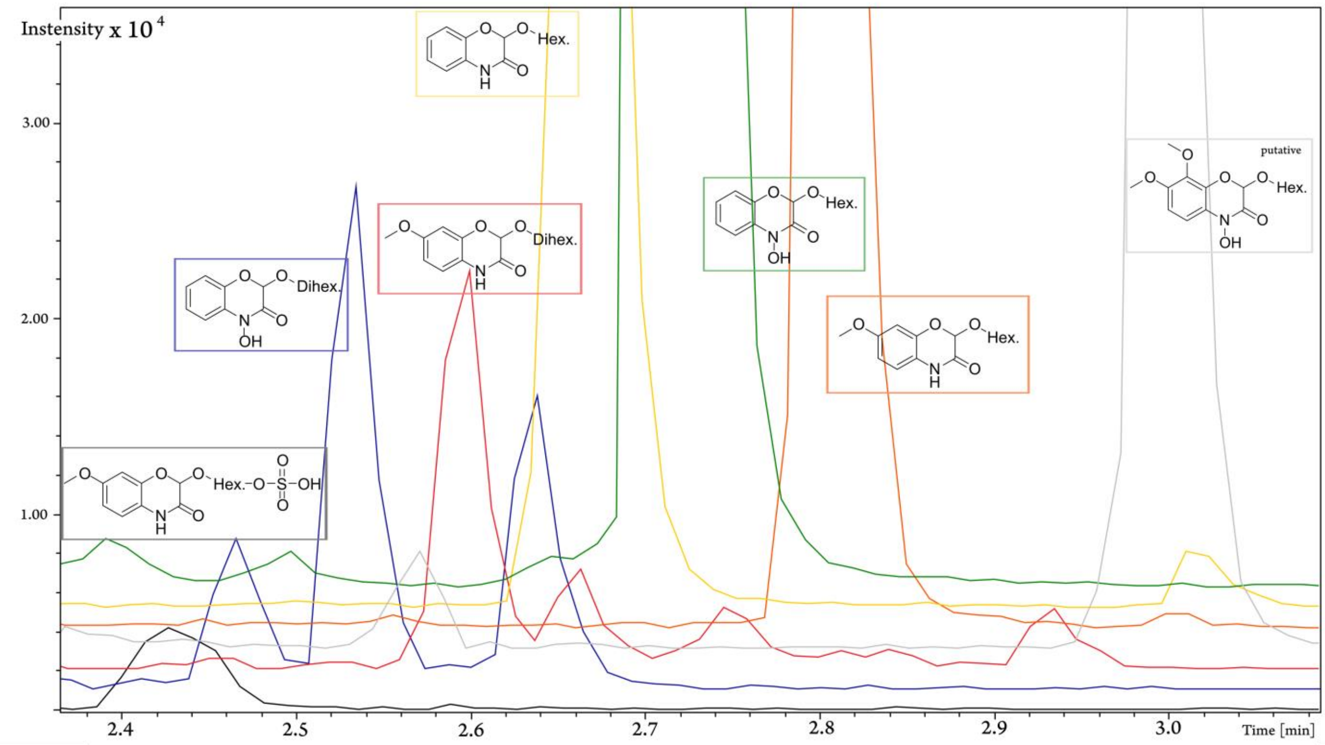

Supplementary Figure 5. Excerpts of the UHPLC-ToF-MS chromatograms of samples 52 and 41. Extracted ion chromatograms of markers for rich hopped beers found by FTICR-MS (blue) found in sample 52 **(A)**. Extracted ion chromatograms of cohulupone (dark blue; confirmed by MS^2 data, compare Table S4) and humulinone isomeric compounds (light blue) **(B)**. Mass traces of identified hops rich beer type markers (compare Table S4) of sample 52 (hops rich craft beer, blue) and sample 41 (wheat beer, green) in comparison **(C)**. Isomeric compounds are shaded blue (for sample 52). UHPLC-ToF-MS extracted ion chromatograms (sample 41) of wheat grain marker masses and corresponding structures substantiated by MS^2 data (compare Table S6)**(D)**.

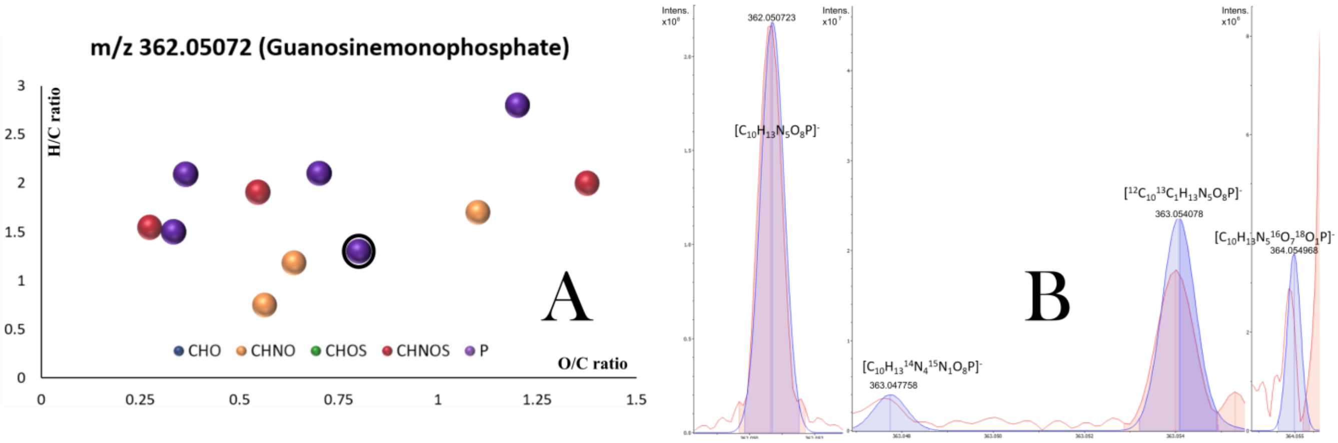

Supplementary Figure 6. Annotation of the mass m/z 362.0572 in context of the underlying available mass error and isotopologue resolution. Eleven valid compositions for m/z 362.05072 in the error window of 3 ppm in a $C_{1\text{-}50}$ $H_{1\text{-}100}$, $O_{0\text{-}50}$ $N_{0\text{-}10}$, $S_{0\text{-}3}$, $P_{0\text{-}1}$, $Cl_{0\text{-}1}$ chemical space **(A)**. Calculations are based on the FormCalc algorithm[14] and restrictions are given by the 'seven golden rules'[15]. The single correct formula inside a 0.1 ppm window is marked. $[C_{10}H_{13}N_5O_8P]^-$ is additionally validated by the isotopic fine structure of the ^{15}N, ^{13}C and ^{18}O isotopologue (beer measurement in red, prediction in blue) **(B)**.

Supplementary References

1 Dresel, M., Vogt, C., Dunkel, A. & Hofmann, T. The bitter chemodiversity of hops (*Humulus lupulus* L.). *J. Agric. Food Chem.* **64**, 7789-7799 (2016).

2 Caspi, R. *et al.* The MetaCyc database of metabolic pathways and enzymes. *Nucleic Acids Res.* **46**, 633-639 (2018).

3 Borremans, F., De Potter, M. & De Keukeleire, D. Carbon-13 NMR spectroscopy of hop bitter substances. *Org. Magn. Reson.* **7**, 415-417 (1975).

4 Vogt, C. *Strukturanalytische Studien zu bitteren, antimikrobiellen und schaumstabilisierenden Hopfeninhaltsstoffen und Oxidationsprodukten in Bier*, Technical University of Munich (TUM), (2015).

5 Intelmann, D. *Molekulare, psychophysikalische und rezeptorbasierte Studien zum Bittergeschmack von Bier*, Technical University of Munich (TUM), (2010).

6 Kowaka, M., Kokubo, E. & Kuroiwa, Y. New bitter substances of beer: lupoxes c and lupdoxes b. *Proc. Am. Soc. Brew. Chem.* **30**, 42-46 (1972).

7 Haseleu, G. *Sensorische, strukturanalytische und quantitative Studien zu Bitterstoffen aus Hopfen (Humulus lupulus L.) und deren Beitrag zum Bittergeschmack von Bier*, Technical University of Munich (TUM), (2010).

8 Haseleu, G. *et al.* Quantitative sensomics profiling of hop-derived bitter compounds throughout a full-scale beer manufacturing process. *J. Agric. Food Chem.* **58**, 7930-7939 (2010).

9 Intelmann, D., Haseleu, G. & Hofmann, T. LS-MS/MS quantitation of hop-derived bitter compounds in beer using the ECHO Technique. *J. Agric. Food Chem.* **57**, 1172-1182 (2009).

10 Intelmann, D. *et al.* Structures of storage-induced transformation products of the beer's bitter principles, revealed by sophisticated NMR spectroscopic and LC-MS techniques. *Chem. Eur. J.* **15**, 13047-13058 (2009).

11 Pihlava, J.-M. & Kurtelius, T. Determination of benzoxazinoids in wheat and rye beers by HPLC-DAD and UPLC-QTOF MS. *Food Chem.* **204**, 400-408 (2016).

12 Hanhineva, K. *et al.* Qualitative characterization of benzoxazinoid derivatives in whole grain rye and wheat by LC-MS metabolite profiling. *J. Agric. Food Chem.* **59**, 921-927 (2001).

13 de Bruijn, W. J. C., Vincken, J.-P., Duran, K. & Gruppen, H. Mass spectrometric characterization of benzoxazinoid glycosides from rhizopus-elicited wheat (*Triticum aestivum*) seedlings. *J. Agric. Food Chem.* **64**, 6267-6276 (2016).

14 Frommberger, M. *unpublished.*

15 Kind, T. & Fiehn, O. Seven golden rules for heuristic filtering of mulecular formmulas obtained by accurate mass spectrometry. *BMC Bioinformatics* **8** (2007).
